# Repeat domain-associated O-glycans govern PMEL fibrillar sheet architecture

**DOI:** 10.1038/s41598-019-42571-6

**Published:** 2019-04-15

**Authors:** Morven Graham, Athanasia C. Tzika, Susan M. Mitchell, Xinran Liu, Ralf M. Leonhardt

**Affiliations:** 1 Department of Cell Biology, Yale University School of Medicine, 333 Cedar Street, New Haven, CT 06519 USA; 2 Department of Immunobiology, Yale University School of Medicine, 300 Cedar Street, New Haven, CT 06519 USA; 3 Department of Genetics & Evolution, Laboratory of Artificial & Natural Evolution (LANE), Sciences III Building, 1211, Geneva, 4 Switzerland

## Abstract

PMEL is a pigment cell-specific protein that forms a functional amyloid matrix in melanosomes. The matrix consists of well-separated fibrillar sheets on which the pigment melanin is deposited. Using electron tomography, we demonstrate that this sheet architecture is governed by the PMEL repeat (RPT) domain, which associates with the amyloid as an accessory proteolytic fragment. Thus, the RPT domain is dispensable for amyloid formation as such but shapes the morphology of the matrix, probably in order to maximize the surface area available for pigment adsorption. Although the primary amino acid sequence of the RPT domain differs vastly among various vertebrates, we show that it is a functionally conserved, interchangeable module. RPT domains of all species are predicted to be very highly O-glycosylated, which is likely the common defining feature of this domain. O-glycosylation is indeed essential for RPT domain function and the establishment of the PMEL sheet architecture. Thus, O-glycosylation, not amino acid sequence, appears to be the major factor governing the characteristic PMEL amyloid morphology.

## Introduction

**Figure 1:**
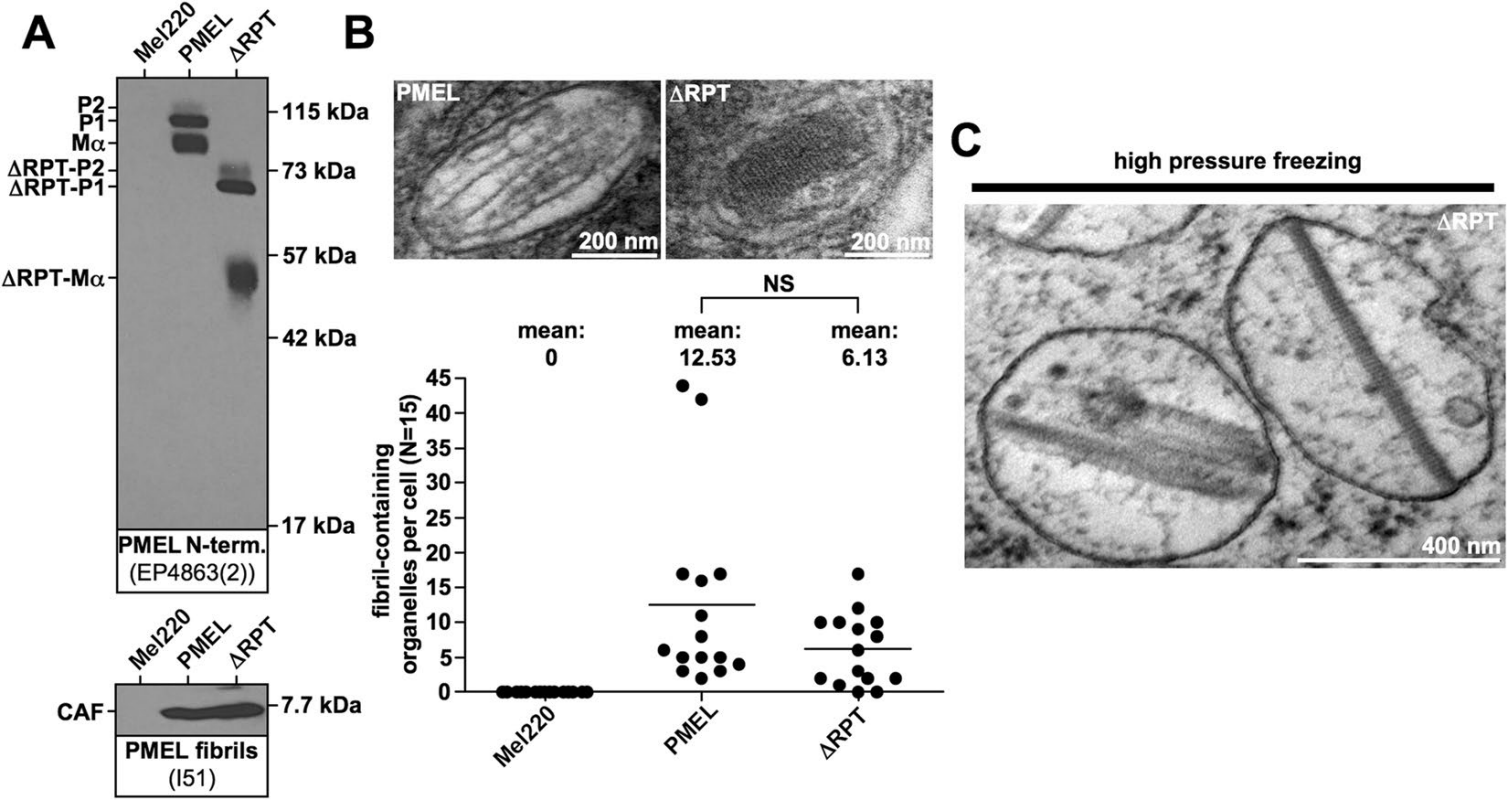
The RPT domain controls the morphology of human PMEL amyloid. (**A**) Western blot analysis of SDS-lysed total membranes using PMEL-specific antibodies EP4863(2) (*PMEL N-term*.) and I51 (*CAF*). (**B**) Conventional TEM and quantitative EM analysis of Mel220 transfectants showing the number of fibril-containing organelles per cell [N = 15]. An unpaired two-tailed t-test was used to determine whether means are statistically different from the wt-PMEL sample (NS, not significant). Representative electron micrographs are depicted. **(C)** EM analysis of ΔRPT-expressing Mel220 cells prepared by high pressure freezing and freeze substitution.

PMEL forms a functional amyloid matrix in melanosomes of pigment cells^1,2^ and is also a potent melanoma antigen^3,4^. The protein assembles into a characteristic architecture of well-separated sheets consisting of laterally associated amyloid fibrils^5^. The resulting matrix serves for the deposition of the pigment melanin and likely also sequesters toxic reaction intermediates in the melanin synthesis pathway^6^, keeping these from diffusing out of the organelle and into the cell^2^. The amyloid core of the fibrils is formed by a proteolytic product derived from the PMEL protein^1^. This proteolytic fragment assembling into and forming the core was originally discovered via its reactivity with antibody I51^7^. We termed this product the core amyloid fragment (CAF) and recently unraveled its sequence identity^8^. There is a second type of proteolytic fragment associated with melanosomal fibrils, MαC^9^, which is also derived from the PMEL protein and which contains the polycystic kidney disease (PKD) domain and the highly O-glycosylated repeat (RPT) domain^8,9^. After incorporation into the fibrils, this fragment undergoes massive proteolysis both within the RPT domain as well as between the PKD domain and the RPT domain^9^. The PKD domain is essential for fibril formation^9,10^ and may possibly contribute to the fibril core alongside the CAF^7^. The RPT domain had originally been suggested to be required for amyloid formation based on studies in HeLa cells^9,10^ and was even proposed to form the amyloid core^11,12^. However, later work showed that this domain is dispensable for amyloid formation both *in vitro*^7^ as well as *in vivo* in living melanocytic cells^13^. However, the RPT domain controls amyloid morphology, as the characteristic PMEL sheet structure appears disrupted in melanoma cells expressing a PMEL-ΔRPT deletion mutant^13^ (Fig. 1). Nevertheless, precisely how the amyloid structure is altered in the absence of the repeat domain is difficult to understand from electron microscopy (EM) two-dimensional imaging. This is an important problem, because the shape of the matrix is tightly linked to its function, as its organization into sheets maximizes the surface area available for melanin deposition. Moreover, the mechanism by which the sheet architecture is established is unknown; a problem that is confounded by the puzzling fact that the primary amino acid sequence of the RPT domain displays very high evolutionary divergence. Here, we use electron tomography to resolve the three-dimensional architecture of PMEL amyloid formed in the absence of the highly glycosylated RPT domain. We show that without this domain, the characteristic sheet structure of the melanosomal amyloid collapses and that maintaining the sheet architecture requires O-glycosylation. We also demonstrate that the RPT domain, despite differing dramatically in amino acid sequence between species, is a functional module, which is largely interchangeable between PMEL orthologues.

## Results

### The RPT domain is required for normal melanosomal amyloid morphology

PMEL is a transmembrane glycoprotein that is exported from the endoplasmic reticulum (ER) in an immature form called P1. Along the secretory route, N-glycans undergo modifications and extensive O-glycosylation is added, resulting in a significant shift in the apparent molecular weight, giving rise to the so-called P2 form. While migrating to the plasma membrane the protein is cleaved by furin, which produces two cleavage products, the membrane-standing C-terminal Mβ fragment and the lumenal N-terminal Mα fragment^14–17^. These remain linked to each other by a disulfide bridge involving Cys-301^18^. Because the cellular levels of the P2 form are usually negligible, the steady state P1:Mα or P1:Mβ ratios (ER form versus post-ER forms) are good measures of the ER export efficiency, which depends on proper protein folding^13,19^.

Based on this criterion, a human PMEL mutant lacking most of the RPT domain, ΔRPT (Δ315–431)^13^, stably expressed in the PMEL-deficient human melanoma cell line Mel220, folded well and was efficiently released from the ER (Fig. 1A, *top panel*). At steady state, we also found similar levels of the core amyloid fragment (CAF) accumulating in cells expressing wildtype human PMEL and the corresponding ΔRPT mutant (Fig. 1A, *bottom panel*). Because the CAF is extremely unstable in cells unless it undergoes incorporation into melanosomal amyloid^13^, all CAF detectable by Western blotting likely represents amyloid-associated material. Hence, consistent with our previous results^13^, the RPT domain is not required for amyloid formation. This result was confirmed by electron microscopy (EM), which detected vigorous, albeit somewhat reduced fibril formation for the mutant (Fig. 1B). However, as described previously^13^, ΔRPT-mutant amyloid had an altered, block-like morphology, apparently lacking the characteristic sheet structure^5^ observed with the wildtype protein (Fig. 1B). This phenotype was the same irrespective of whether cells had been prepared by conventional fixation (Fig. 1B) or by high pressure freezing (Fig. 1C).

### Mouse PMEL is fully functional and behaves like human PMEL in Mel220 cells

**Figure 2:**
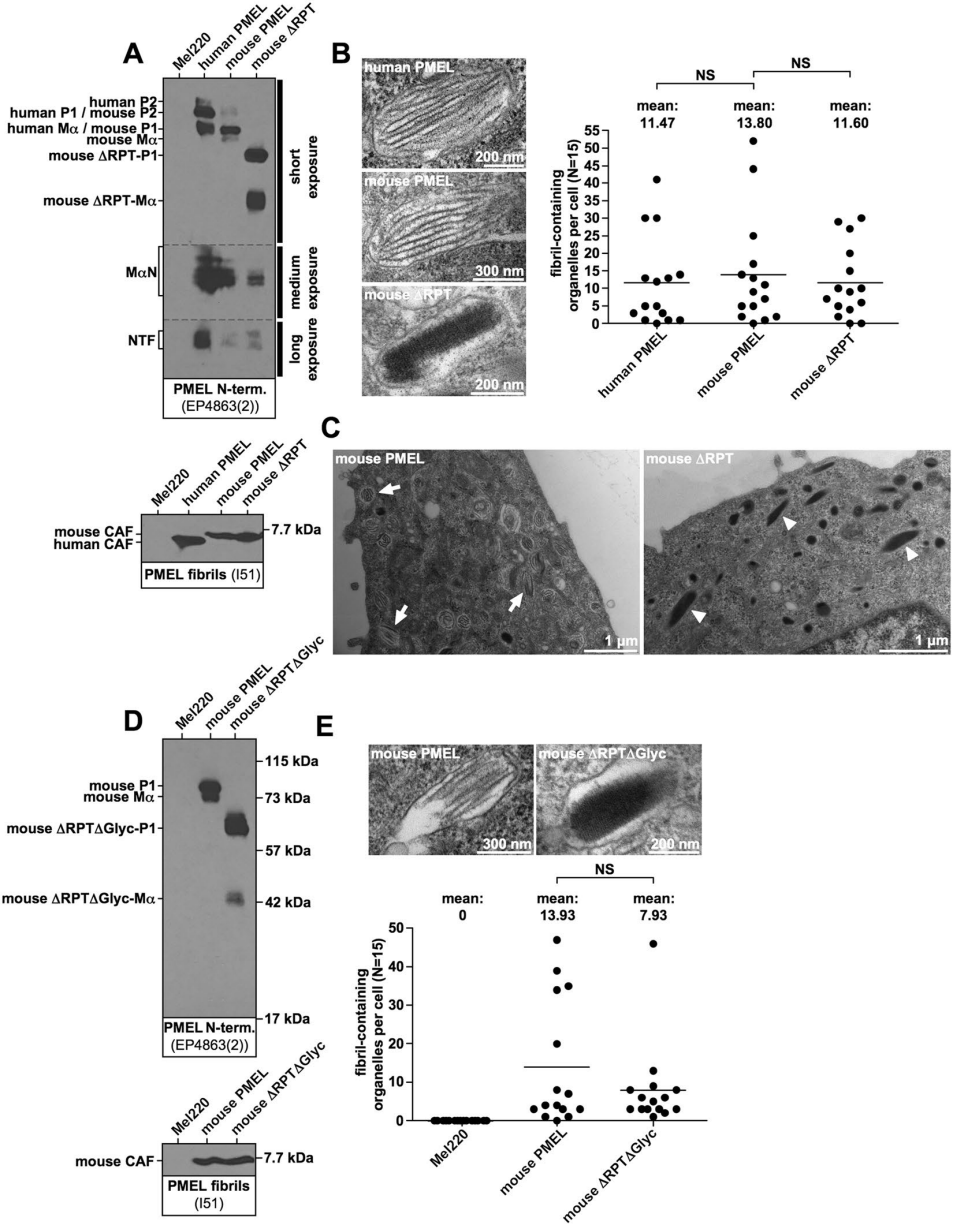
The RPT domain controls the morphology of murine PMEL amyloid. (**A**,**D**) Western blot analysis of SDS-lysed total membranes using PMEL-specific antibodies EP4863(2) (*PMEL N-term*.) and I51 (*CAF*). **(B**,**E)** Quantitative EM analysis of Mel220 transfectants showing the number of fibril-containing organelles per cell [N = 15]. Fig. 2E also shows untransduced, PMEL-free Mel220 cells as a negative control. A One-Way ANOVA with Dunnett’s post test (**B**) or an unpaired two-tailed t-test (**E**) were used to determine whether means are statistically different from the mouse PMEL sample (NS, not significant). Representative electron micrographs are depicted. (**C**) Lower magnification image of the EM analysis in (**B**). Arrows point to melanosomes with regular sheet morphology. Arrowheads point to abnormal melanosomes containing block-like collapsed amyloid.

Wildtype mouse PMEL was expressed in Mel220 cells at levels comparable to the human protein (Fig. 2A, *top panel*) and formed fibrils that were quantitatively and qualitatively indistinguishable from human PMEL (Fig. 2A, *bottom panel*, and Fig. 2B). Thus, mouse PMEL is fully functional in human cells. Like its human counterpart described in Fig. 1A, a comparable mouse ΔRPT mutant (Δ315–402) displayed the same abnormal, block-like amyloid morphology (Fig. 2B,C), indicating that the RPT domain plays the same role in mouse and human PMEL. Again, we observed no significant quantitative defect in fibril formation, neither on the level of CAF accumulation (Fig. 2A, *bottom panel*) nor in the overall number of fibril-containing organelles per cell (Fig. 2B).

The RPT domain of human and mouse PMEL consists of 12 and 10 imperfect repeats of 13 amino acids, respectively, but the mutants assessed in Fig. 1 and Fig. 2A–C retain the first and the two last repeats. To exclude that PMEL fibril formation requires these outmost repeats, we constructed a mouse PMEL mutant with a more extensive deletion, Δ304–429, in which additionally three potentially O-glycosylated residues (Ser-299, Ser-302, and Ser-303) were mutated. The resulting mutant was called ΔRPTΔGlyc (Δ304–429/S299G/S302P/S303G) and fully lacks the entire RPT domain. The mutant also completely lacks any predicted O-glycosylation within MαC. This mutant was expressed in Mel220 cells, matured normally as judged by the P1:Mα ratio at steady state (Fig. 2D, *top panel*), and was quantitatively largely unaffected in fibril formation (Fig. 2D, *bottom panel*, and Fig. 2E). This confirms that no part of the RPT domain is required to form melanosomal amyloid in cells. However, like other ΔRPT mutants, construct ΔRPTΔGlyc formed abnormal, block-like assemblies devoid of the characteristic sheet structure (Fig. 2E).

### The PMEL fibrillar sheet structure collapses in the absence of the RPT domain

**Figure 3:**
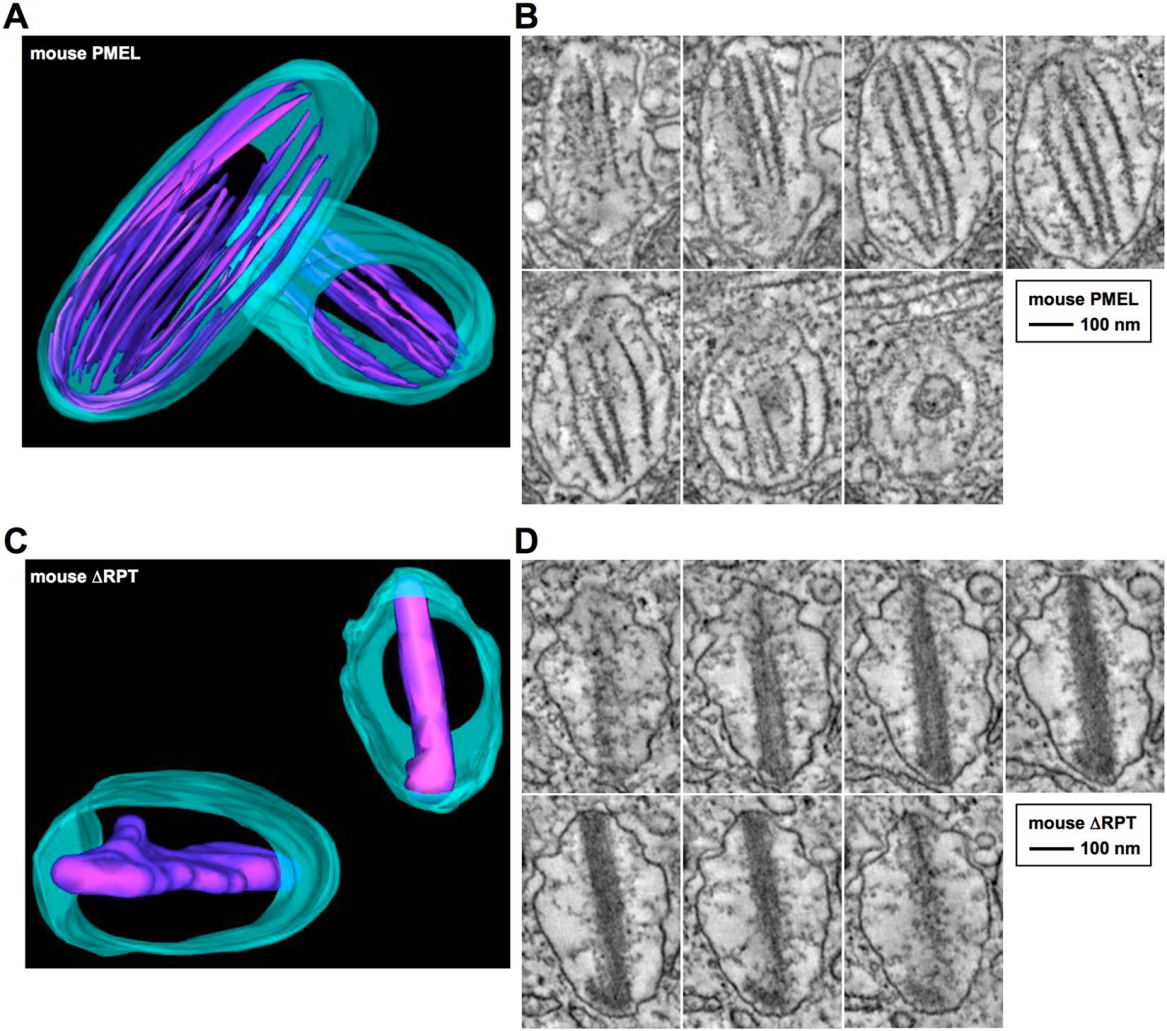
The sheet structure of the PMEL amyloid collapses in the absence of the RPT domain. (**A**,**C**) Three-dimensional electron tomography models of representative melanosomes in different Mel220 transfectants. Parts of the tomographic tilt series underlying these models are shown in (**B**,**D**). The melanosomal limiting membrane is shown in turquoise. The amyloid is shown in violet. (**B**,**D**) Representative sequential slice views of the tomographic tilt series underlying the 3D models shown in (**A**,**C**). The organelle shown in (**B**) corresponds to the lower left organelle in (**A**). The organelle shown in (**D**) corresponds to the upper right organelle in (**C**).

To gain insights into how the RPT domain shapes the three-dimensional architecture of melanosomal amyloid, we resorted to electron tomography. This technique had previously been used to demonstrate the structural organization of human PMEL fibrillar material into sheets^5^. In line with this earlier work^5^, mouse PMEL expressed in Mel220 cells also assembled into well-separated amyloid sheets with a thickness of ∼7–12 nm (Fig. 3A,B, Movie S1). These sheets spanned the entire length of the organelle (535 nm across the long axis of the melanosome in Fig. 3B). In stark contrast, mouse ΔRPT developed no sheet architecture, with the entire amyloid essentially collapsing into a dense block of material (Fig. 3C,D, Movie S2). A regular, repetitive pattern perpendicular to the long fibril axis was often visible, further underscoring the nature of these assemblies as amyloid (Fig. 2B, *bottommost panel*, and Fig. 3D). Notably, ΔRPT amyloid also spanned the entire organelle (480 nm across the long axis of the melanosome in Fig. 3D), suggesting that its morphological defect is limited to an altered organization of the material along the short axis of the organelle. In contrast, there is no evidence that fibril extension along the long axis or lateral fibril assembly is affected. Thus, the main function of the RPT domain appears to be preventing the collapse of the PMEL fibrillar sheet architecture into a single block-like mass.

### Extensive O-glycosylation not primary amino acid sequence appears to be the conserved feature of the RPT domain

**Figure 4:**
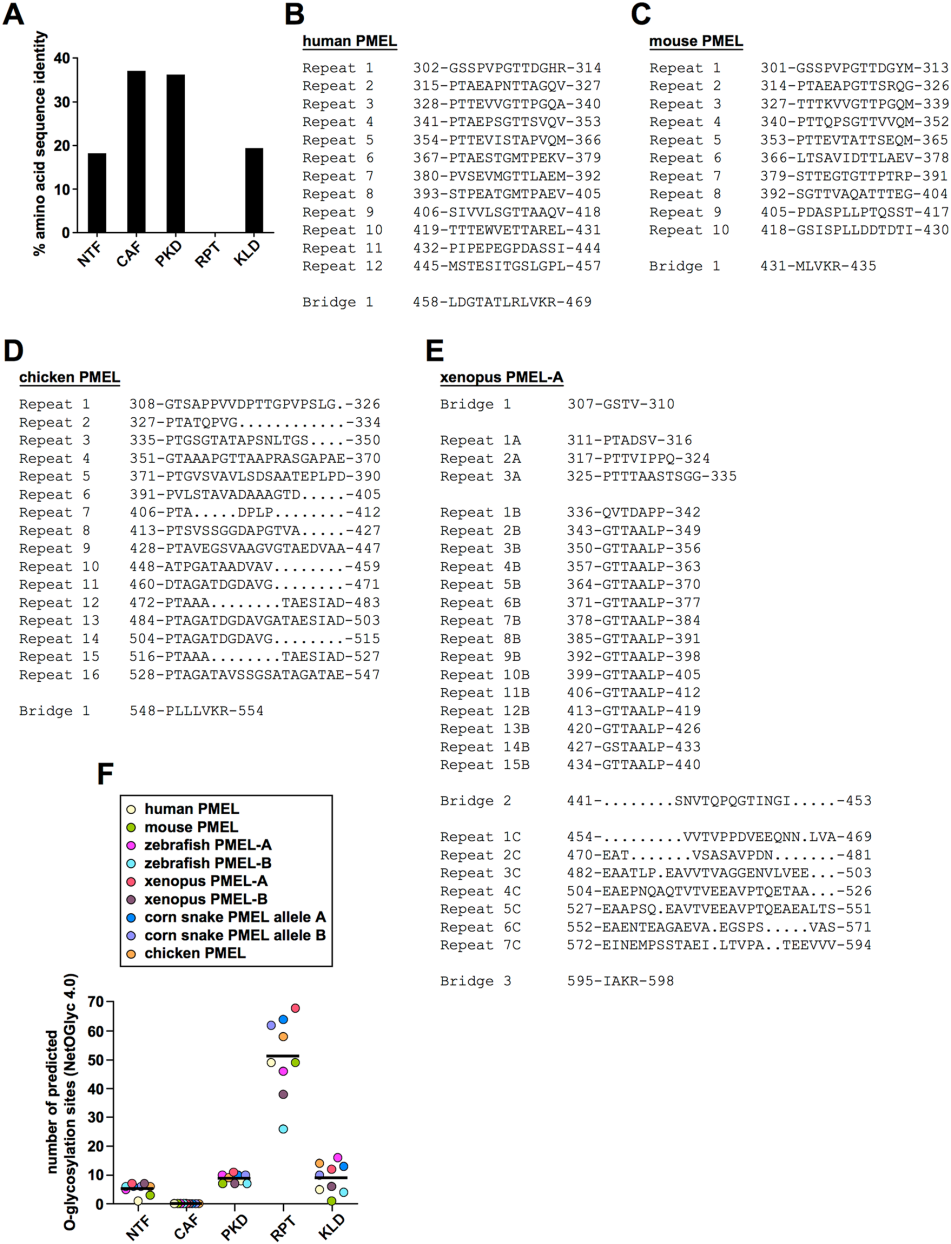
Extensive O-glycosylation not conserved primary amino acid sequence is the shared hallmark of all RPT domains. (**A**) Percent amino acid identity in the major PMEL lumenal domains based on the multiple sequence alignment shown in Supplementary Fig. S1A. **(B**–**E)** Selected RPT domain sequences of human (**B**), mouse (**C**), chicken (**D**), and frog (**E**) origin. For the purpose of this study, the RPT domain is defined as the region within PMEL ranging from the first amino acid following Cys-301 in human PMEL (or the corresponding cysteine residue in non-human PMEL) up until and including the full proprotein convertase cleavage motif. **(F)** Prediction of the number of O-glycosylation sites by the NetOGlyc 4.0 algorithm in the individual lumenal domains of the indicated PMEL genes.

Given that the RPT domain is critical in shaping the PMEL amyloid, and the morphology of the matrix is likely crucial for its function, it is baffling that the primary amino acid sequence of this domain is not conserved^20^ (Fig. 4A and Supplementary Fig. S1A). In fact, neither the organization, nor the number, nor the colinearity of repeats is conserved (Fig. 4B–E). For instance, while mouse and human repeats are similar in nature and fully colinear, they differ in number (Fig. 4B,C). Chicken PMEL also differs from both in repeat number, but repeats additionally lack colinearity, presenting with various gaps (Fig. 4D). In fact, chicken repeats appear to be based on an internal three-segment structure (a-b-c). Segment a (consensus: PTAGA) provides an almost invariant proline-threonine sequence, which is predicted to be O-glycosylated. Segment b (consensus: TxGDAxxx, x = any amino acid) is less conserved, typically provides further serine/threonine residues and frequently contains a negative charge. Segment c (consensus: TAESΨA[−]; Ψ = L, I, V; [−] = D, E) almost invariably contains negative charge(s) as well as further residues predicted to be O-glycosylated. While some repeats feature the complete a-b-c motif (*e.g*. repeats 9 and 16), others lack one or two segments (*e.g*. repeats 10 and 11 with a shortened a-b structure or repeats 12 and 15 with a shortened a-c structure) (Fig. 4D). This potentially has important structural consequences, because - based on modeling and the regular repeat organization of the human RPT domain - others had speculated that the region may attain a β-solenoid structure forming amyloid independently of the CAF^21^. The chicken RPT domain would be very hard to imagine to assemble into a similar architecture, given its irregularly interrupted and incomplete repeats. Frog PMEL repeats have a strikingly different organization when compared to mammals or chickens, displaying different types of repeats (15 near identical repeats of a seven amino acid sequence followed by a more loosely repetitive acidic region dominated by glutamate) (Fig. 4D). The snake PMEL RPT domain barely features obvious recognizable repeats (Supplementary Fig. S1A).

Because the human PMEL RPT domain is thought to be extensively O-glycosylated^22,23^, we wondered whether O-glycosylation may be the shared feature of RPT domains in all species. To address this, we used NetOGlyc 4.0^24^ to predict the number of mucin-type GalNAc O-glycosylation sites in the RPT domains of mammalian, bird, reptile, amphibian, and fish PMEL. Interestingly, for all species examined the RPT domain was predicted to be extensively O-glycosylated (Fig. 4F). This was in contrast to all other major lumenal domains of the protein, which were predicted to contain no or substantially fewer O-glycosylation sites (Fig. 4F). Taken together, not primary amino acid sequence or domain organization but extensive O-glycosylation appears to be the common feature of PMEL RPT domains.

### The RPT domains derived from reptile, amphibian, and bird species are functional in human cells

**Figure 5:**
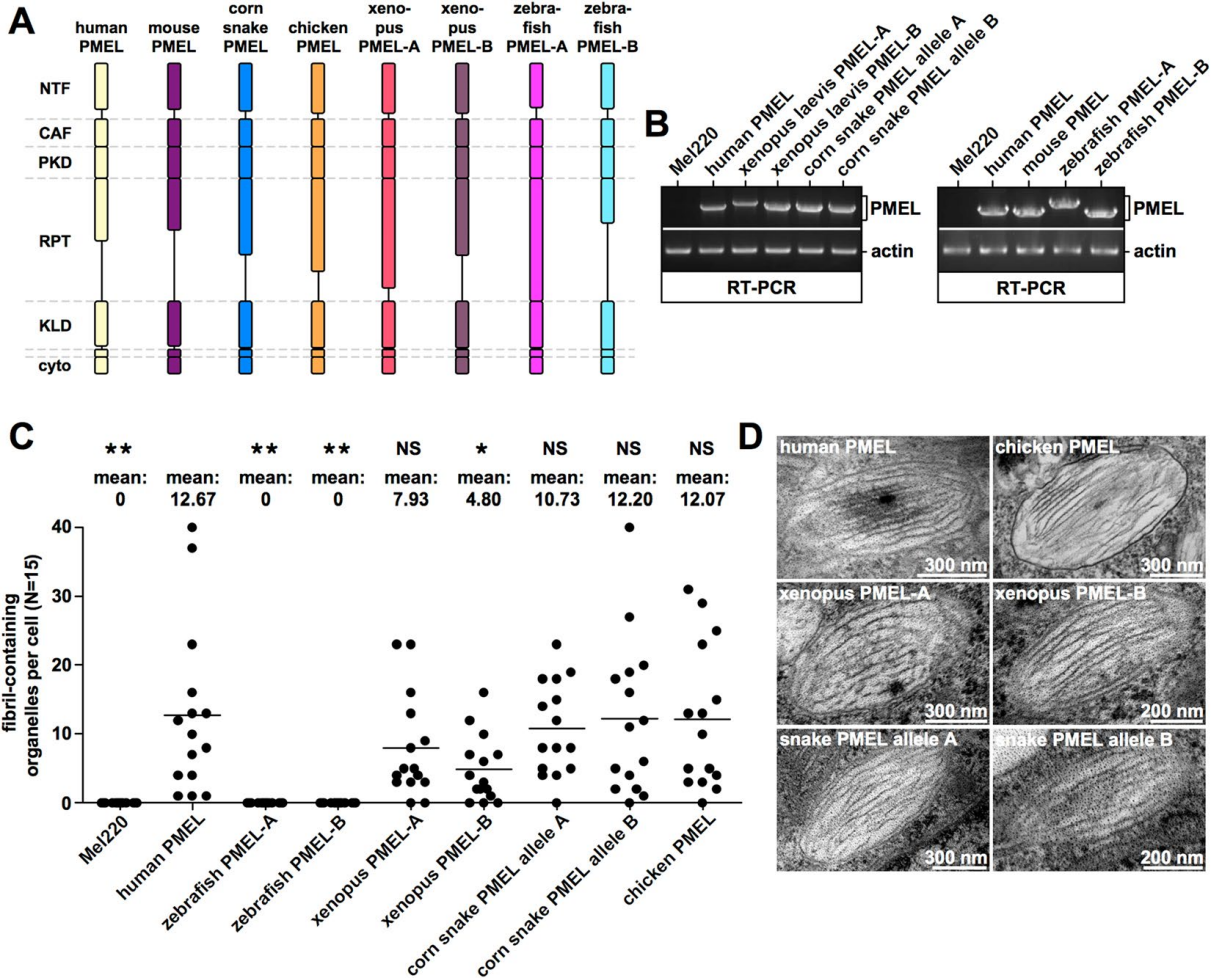
Fibril formation by non-human PMEL genes in human Mel220 cells. (**A**) Schematic representation of the various human and non-human PMEL genes analyzed. Note that RPT domain length, but not the length of any of the other major domains, varies dramatically between species. (**B**) Confirmation of PMEL construct expression in Mel220 cells by semi-quantitative RT-PCR. The primers used are vector-specific primers amplifying the entire PMEL open reading frame. The same primers were used for all constructs to allow cross-comparability. (**C**,**D**) Quantitative EM analysis of Mel220 transfectants showing the number of fibril-containing organelles per cell [N = 15]. A One-Way ANOVA with Dunnett’s post test was used to determine whether means are statistically different from the human PMEL sample (*p < 0.05; **p < 0.01; NS, not significant). Representative electron micrographs are depicted (**D**).

Given that the primary amino acid sequence of the RPT domain is not conserved across species, we asked whether these domains nevertheless share the same function. To address this, we focused on a set of PMEL molecules derived from fish, frog, snake, and chicken (Fig. 5A). Some of these (zebrafish PMEL-A and PMEL-B as well as Xenopus laevis PMEL-A and PMEL-B) were readily commercially available. For chicken PMEL only the pathogenic *Dominant white* PMEL allele^25,26^ was commercially available, from which we removed the three amino acid insertion (695-WAP-697) in the transmembrane domain to produce the functional wildtype allele. In order to obtain reptile PMEL, we cloned from one individual corn snake two alleles of the single snake PMEL gene. The two alleles differ from each other by five SNPs, one of which is silent (Supplementary Fig. S2). All non-silent SNPs are located inside the RPT domain (Supplementary Fig. S2D).

PMEL genes from the different species were stably transduced into Mel220 cells and their expression was assessed by RT-PCR. Surprisingly, chicken PMEL expression was not detected in this assay (*data not shown*), although the gene is almost certainly expressed (see below). The problems in detection may reflect the significantly higher GC content of chicken PMEL (68% versus 48–56% in all other species), potentially resulting in more stable nucleic acid secondary structures interfering with cDNA synthesis. Accordingly, unlike with other PMEL genes, we also experienced substantial problems to sequence through certain regions of the chicken PMEL cDNA (*data not shown*). In any case, the expression of all other foreign PMEL genes as well as human PMEL was readily detected by RT-PCR (Fig. 5B). When assessed by EM, neither zebrafish PMEL-A nor zebrafish PMEL-B formed any fibrils in two independent experiments (Fig. 5C and *data not shown*), indicating that fish PMEL is not functional in human cells. This result was particularly surprising with respect to zebrafish PMEL-A, which in contrast to its paralog PMEL-B has been shown to form functional melanosomal amyloid in fish retinal pigment epithelium (RPE)^27,28^. Nevertheless, all other PMEL proteins including chicken PMEL did form melanosomal amyloid (Fig. 5C,D). Fibril formation was slightly reduced in case of the two frog PMEL paralogs, but this was statistically significant only for Xenopus PMEL-B (Fig. 5C). Most importantly, fibril morphology was normal in all cases with well-separated fibril sheets (Fig. 5D). Given that this morphology is governed by the RPT domain (Figs 1–3), this strongly suggests that despite dramatic differences in sequence (Fig. 4A–E and Supplementary Fig. S1A) all RPT domains are functionally equivalent.

### RPT domain function is conserved across species

**Figure 6:**
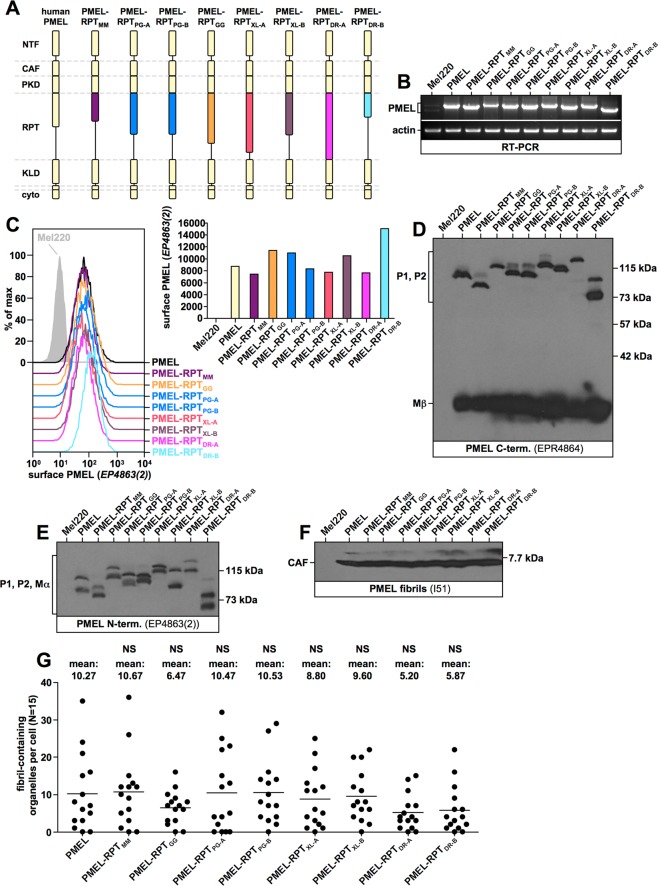
Fibril formation by PMEL RPT domain swapping mutants. (**A**) Schematic representation of the various chimeric PMEL mutants analyzed. Mutants are based on human PMEL and contain the RPT domain from mouse PMEL (PMEL-RPT_MM_), corn snake PMEL allele A (PMEL-RPT_PG-A_), corn snake PMEL allele B (PMEL-RPT_PG-B_), chicken PMEL (PMEL-RPT_GG_), xenopus laevis PMEL-A (PMEL-RPT_XL-A_), xenopus laevis PMEL-B (PMEL-RPT_XL-B_), zebrafish PMEL-A (PMEL-RPT_DR-A_), or zebrafish PMEL-B (PMEL-RPT_DR-B_). (**B**) Confirmation of PMEL construct expression in Mel220 cells by semi-quantitative RT-PCR. The primers used are vector-specific primers amplifying the entire PMEL open reading frame. The same primers were used for all constructs to allow cross-comparability. **(C)** Flow cytometry analysis of the surface expression of PMEL chimeric constructs. Results are depicted as histograms (*left panel*) or in form of a bar diagram (*right panel*). **(D**–**F)** Western blot analysis of SDS-lysed total membranes using PMEL-specific antibodies EPR4864 (*PMEL C-term*.) (**D**), EP4863(2) (*PMEL N-term*.) (**E**), and I51 (*CAF*) (**F**). Lanes in Fig. 6D contain three bands (a highly intense, low molecular weight form corresponding to the C-terminal Mβ fragment (∼27 kDa), an intermediate intensity, middle band corresponding to the immature ER form P1 (∼100 kDa in human PMEL), and a topmost weak band, running slightly slower and corresponding to the Golgi form P2 (∼120 kDa in human PMEL). P2 is not visible for all constructs in this exposure. Lanes in Fig. 6E also contain three bands. The lowest molecular weight form corresponds to the N-terminal Mα fragment (∼85 kDa in human PMEL), the middle band corresponds to P1, and the topmost weak band corresponds to P2. P2 is not visible for all constructs in this exposure. **(G)** Quantitative EM analysis of Mel220 transfectants showing the number of fibril-containing organelles per cell [N = 15]. A One-Way ANOVA with Dunnett’s post test was used to determine whether means are statistically different from the human PMEL sample (NS, not significant). Representative electron micrographs are depicted in Fig. 7B.

To assess whether RPT domains from different species are functionally interchangeable, we cloned various human PMEL constructs, in which the RPT domain was substituted for a foreign RPT domain from mouse (MM, *Mus musculus*), chicken (GG, *Gallus gallus*), snake (PG, *Pantherophis guttatus*), frog (XL, *Xenopus laevis*), or fish (DR, *Danio rerio*) origin (Fig. 6A). All constructs were stably expressed in Mel220 cells and their expression was confirmed by RT-PCR (Fig. 6B). Moreover, like wildtype human PMEL, all chimeric constructs were detected at the cell surface (Fig. 6C), where the molecules transiently appear before they migrate further into early melanosomes^15,29^. P1:Mβ (Fig. 6D) and P1:Mα (Fig. 6E) ratios measured by Western blotting indicated that all constructs folded well and were efficiently exported from the ER. Furthermore, all constructs were comparably active in forming fibrils, as judged by CAF accumulation (Fig. 6F) and direct analysis by EM (Fig. 6G).

**Figure 7:**
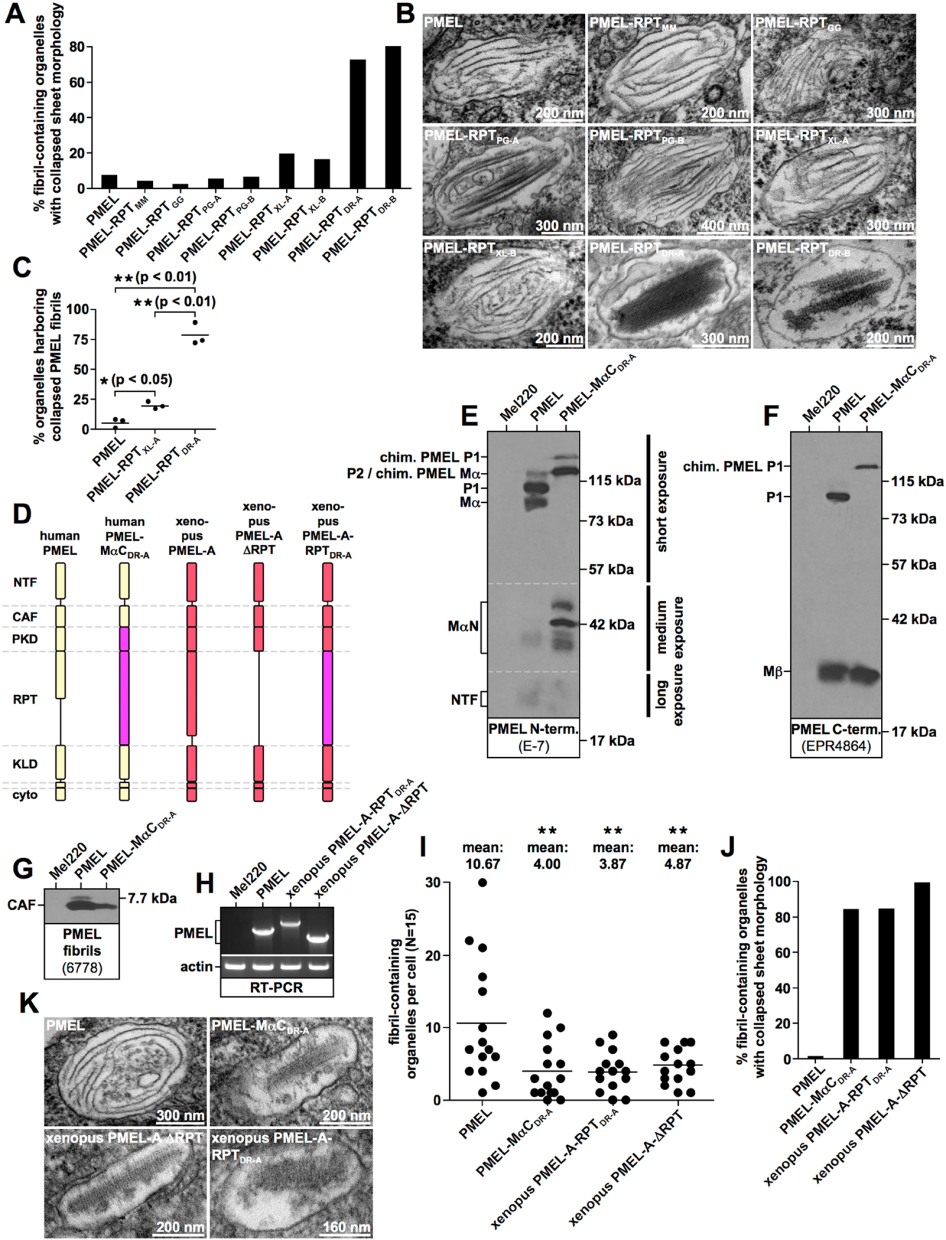
Amyloid morphology in Mel220 cells expressing RPT-chimeric PMEL mutants. (**A**,**J**) Quantification of the number of fibril-containing organelles displaying the collapsed amyloid sheet phenotype. Results shown in Fig. 6G and (**A**,**B**) are derived from the same experiment. (**B**,**K**) Representative electron micrographs corresponding to the experiment in Fig. 6G and (**A**,**B**) as well as to the experiment in (**I**,**J**,**K**), respectively. **(C**) Quantification of the number of fibril containing organelles displaying the collapsed amyloid sheet phenotype. Shown is the average from three independent experiments including the experiment shown in (**A**). A One-Way ANOVA with Dunnett’s post test was used to determine whether means are statistically different from the human PMEL sample (*p < 0.05; **p < 0.01). (**D**) Schematic representation of chimeric PMEL mutants. Construct PMEL-MαC_DR-A_ is based on human PMEL and contains the entire MαC region from zebrafish PMEL-A. Construct xenopus PMEL-A-RPT_DR-A_ is based on xenopus laevis PMEL-A and contains the RPT domain from zebrafish PMEL-A. **(E**–**G**) Western blot analysis of SDS-lysed total membranes using PMEL-specific antibodies E-7 (*PMEL N-term*.) (**E**) EPR4864 (*PMEL C-term*.) (**F**) and 6778 (*CAF*) (**G**). **(H)** Confirmation of PMEL construct expression in Mel220 cells by semi-quantitative RT-PCR. The primers used are vector-specific primers amplifying the entire PMEL open reading frame. The same primers were used for all constructs to allow cross-comparability. (**I**) Quantitative EM analysis of Mel220 transfectants showing the number of fibril-containing organelles per cell [N = 15]. A One-Way ANOVA with Dunnett’s post test was used to determine whether means are statistically different from the human PMEL sample (**p < 0.01). Representative electron micrographs are depicted in (**K**). Melanosome morphology is quantified in (**J**).

**Figure 8:**
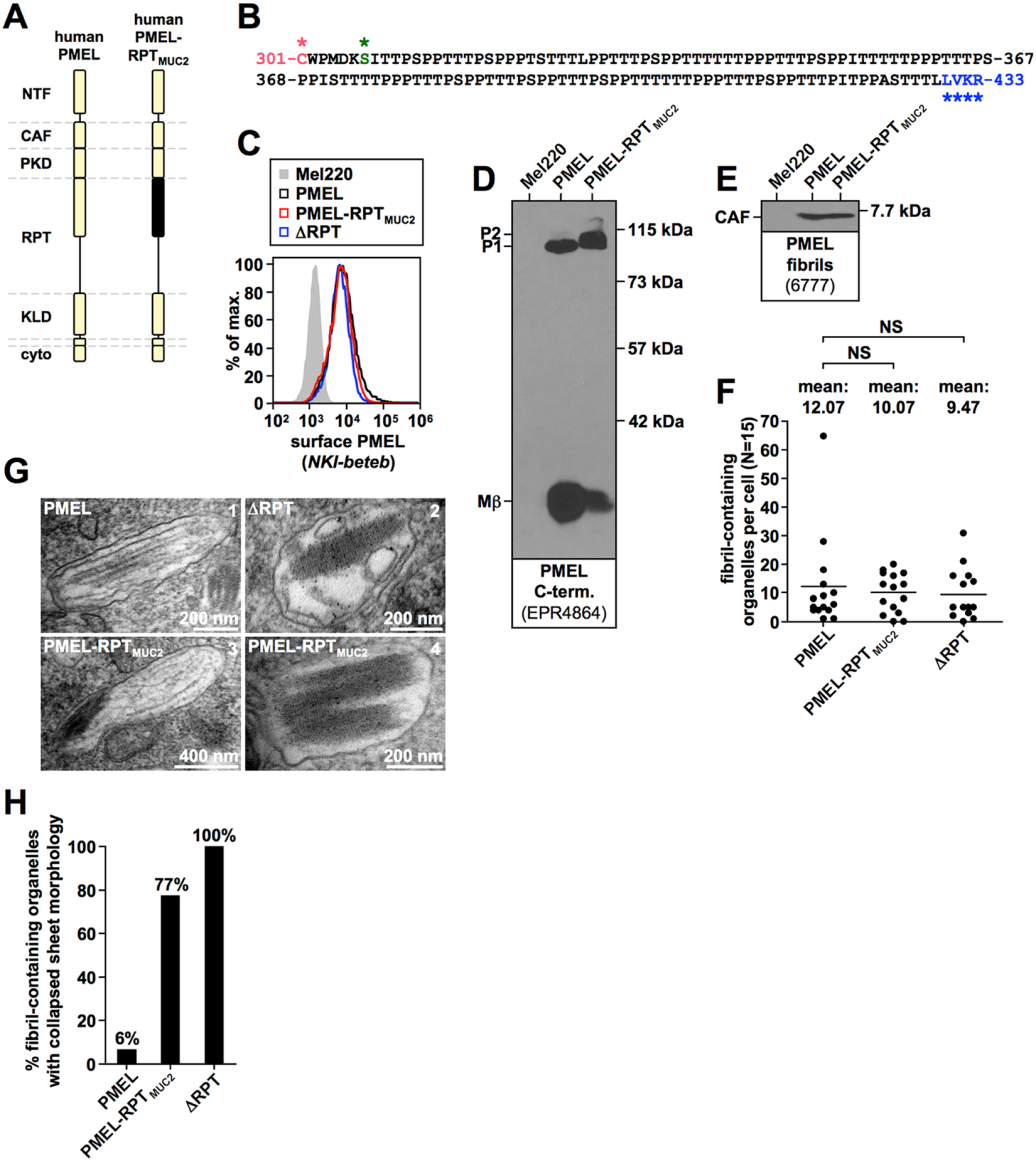
A randomly selected, O-glycosylated segment from MUC2 can partially substitute for the human RPT domain. (**A**) Schematic representation of the MUC2-chimeric PMEL construct PMEL-RPT_MUC2_. (**B**) Amino acid sequence of the MUC2 segment contained in PMEL-RPT_MUC2_. NetOGlyc 4.0 predicts 71 O-glycosylation sites within the MUC2 segment. **(C)** Flow cytometry analysis of the surface expression of human PMEL, human ΔRPT, and chimeric construct PMEL-RPT_MUC2_. **(D**,**E**) Western blot analysis of SDS-lysed total membranes using PMEL-specific antibodies EPR4864 (*PMEL C-term*.) (**D**) and 6777 (*CAF*) (E). **(F,G**) Quantitative EM analysis of Mel220 transfectants showing the number of fibril-containing organelles per cell [N = 15]. A One-Way ANOVA with Dunnett’s post test was used to determine whether means are statistically different from the wildtype human PMEL sample (NS, not significant). Representative electron micrographs are depicted (**G**). Note that some organelles in PMEL-RPT_MUC2_-expressing cells contain well-separated sheets (*panel 3*), which are never observed in ΔRPT-expressing cells (Supplementary Fig. S4H). **(H)** Quantification of the number of fibril containing organelles displaying the collapsed amyloid sheet phenotype.

Strikingly, the highly divergent RPT domains from mouse (PMEL-RPT_MM_), chicken (PMEL-RPT_GG_), and snake PMEL (PMEL-RPT_PG-A_ (allele A) and PMEL-RPT_PG-B_ (allele B)) were all able to fully substitute for the function of the human domain. Less than 7% of melanosomes harbored fibrils displaying the collapsed sheet phenotype characteristic of ΔRPT mutants (Fig. 7A,B). This is within the range of what is observed for human PMEL (Fig. 7A). Interestingly, RPT domains from Xenopus PMEL-A (PMEL-RPT_XL-A_) and PMEL-B (PMEL-RPT_XL-B_) were slightly less efficient in promoting proper sheet separation (19% and 16% organelles with collapsed sheet phenotype, respectively) (Fig. 7A) and this small difference was statistically significant in three independent experiments (Fig. 7C). Nevertheless, also the frog RPT domains were largely functional with more than 80% of melanosomes displaying the normal morphology (Fig. 7A–C and Supplementary Fig. S3A–D). Surprisingly, the RPT domains from zebrafish PMEL-A (PMEL-RPT_DR-A_) and PMEL-B (PMEL-RPT_DR-B_) were much less efficient in substituting for the function of the human domain (Fig. 7A,B and Supplementary Fig. S3A–D). 72% and 80% of melanosomes displayed the collapsed sheet phenotype, respectively (Fig. 7A,B), and this result was highly statistically significant (Fig. 7C). Nevertheless, we note that even the fish RPT domains were at least partially functional in the context of the human protein, because ΔRPT mutants lacking the repeat domain altogether display the phenotype of collapsed amyloid sheets in 100% of melanosomes (Fig. 8H).

The fibril-associated MαC fragment consists of the PKD domain and the RPT domain^8,9^. Thus, the fish RPT domain may be less able to form a functional unit with the human PKD domain in construct PMEL-RPT_DR-A_. To address this scenario, we generated a chimeric PMEL construct in which not only the RPT domain, but the entire MαC region in human PMEL was substituted for the corresponding zebrafish PMEL-A sequence (PMEL-MαC_DR-A_) (Fig. 7D). This construct was stably expressed in Mel220 cells and displayed efficient export from the ER, as judged by P1:Mα (Fig. 7E) and P1:Mβ (Fig. 7F) ratios at steady state. The CAF accumulated for the mutant and fibrils were observed by EM, even though to a somewhat reduced extent (Fig. 7G,I). However, construct PMEL-MαC_DR-A_ was similarly impaired in the formation of well-separated fibril sheets (Fig. 7J,K) as construct PMEL-RPT_DR-A_ (Fig. 7A–C). Thus, a pure fish MαC fragment was unable to fully restore RPT domain function in the context of human PMEL. We also attempted to exchange the entire CAF-MαC cassette (encompassing all fibril-associated PMEL fragments) in the human protein for the corresponding fish sequence in order to assess whether a partial incompatibility exists between the fish RPT domain and the human CAF. However, such a construct was not released from the ER in Mel220 cells, indicating misfolding^13^ and precluding further analysis (*data not shown*).

We reasoned that if a potential incompatibility exists between the fish RPT domain and the human CAF, then the fish RPT domain may function more efficiently in the context of a more closely related PMEL ortholog. To test this hypothesis, we generated a PMEL construct based on Xenopus PMEL-A, in which the RPT domain was replaced by the RPT domain derived from zebrafish PMEL-A (Xenopus PMEL-A-RPT_DR-A_) (Fig. 7D). As a control, we generated a Xenopus PMEL-A ΔRPT mutant (Fig. 7D). Both constructs were stably expressed in Mel220 cells (Fig. 7H) and formed fibrils, albeit to a lesser extent than human PMEL (Fig. 7I). As expected, the frog ΔRPT mutant displayed the full collapsed sheet phenotype (Fig. 7J,K). This confirms that mammalian and amphibian RPT domains serve identical functions. However, the Xenopus PMEL-A-RPT_DR-A_ construct showed a similarly inefficient rescue of RPT domain function (Fig. 7J,K) as human PMEL-RPT_DR-A_ (Fig. 7A,C) (84% of organelles displaying the collapsed sheet phenotype). Thus, the fish RPT domain is poorly functional in both human and frog PMEL, perhaps suggesting an intrinsic defect. Alternatively, the fish RPT domain may be functional *per se*, but not efficiently incorporated into human and frog amyloid.

### O-glycosylation not primary amino acid sequence governs the PMEL sheet architecture

Our results in Fig. 4 suggest that O-glycosylation not the primary amino acid sequence is the shared common feature of PMEL RPT domains. In order to directly assess the importance of the RPT domain primary amino acid sequence, we cloned a randomly selected 128 residue sequence predicted to be highly O-glycosylated from human MUC2^30^. The segment was inserted in place of the RPT domain in human PMEL (construct PMEL-RPT_MUC2_) (Fig. 8A,B). The resulting construct was stably expressed in Mel220 cells and was detectable at the cell surface (Fig. 8C), indicating efficient ER export. The P2 form of the mutant was more abundant at steady state than was observed for wildtype PMEL, while Mβ levels were reduced (Fig. 8D). We speculate that this may reflect heavy O-glycosylation right up until the furin cleavage site (Fig. 8B, *blue asterisks*), thereby somewhat reducing cleavage efficiency. In any case, significant levels of Mβ did form (Fig. 8D), indicating that furin cleavage, which is essential for fibril formation^16^, was at least partially functional. Accordingly, the CAF accumulated to normal levels (Fig. 8E) and fibril formation was observed by EM (Fig. 8F). 77% of the melanosomes harbored amyloid material that displayed the collapsed sheet phenotype (Fig. 8G,H), while 23% of the melanosomes displayed the normal morphology with well-separated fibril sheets (Fig. 8G, *panel 3*). As a control, ΔRPT mutants harbored 0% melanosomes with normal sheets (Fig. 8G,H). Thus, the MUC2 sequence, which is completely unrelated to the PMEL repeat regions, can serve as a functional substitute for the human RPT domain with similar efficiency as the corresponding fish RPT domains. That even a randomly selected O-glycosylated sequence is capable of doing this strongly suggests that O-glycosylation, not primary amino acid sequence is the major, defining factor driving RPT domain function.

**Figure 9:**
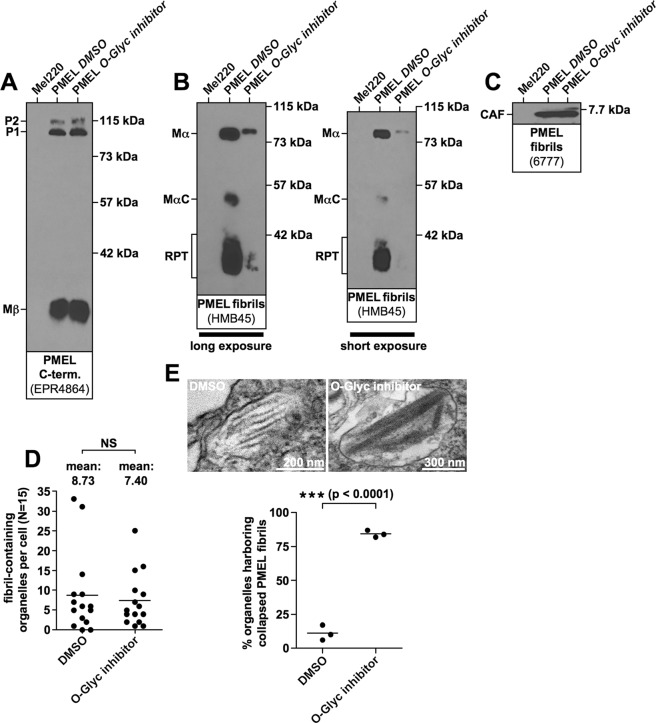
The sheet morphology of melanosomal amyloid essentially depends on functional O-glycosylation. Mel220 transfectants expressing human PMEL were treated for four days with the O-glycosylation inhibitor Benzyl-2-acetamido-2-deoxy-α-D-galactopyranoside or with the solvent DMSO alone. (**A**–**C)** Western blot analysis of SDS-lysed total membranes using PMEL-specific antibodies EPR4864 (*PMEL C-term*.) (A), HMB45 (*sialylated RPT domain*) (B, *two different exposures of the same blot are shown*), and 6777 (*CAF*) (C). (**D**,**E**) Quantitative EM analysis showing the number of fibril-containing organelles per cell [N = 15]. An unpaired two-tailed t-test was used to determine whether means are statistically different from the DMSO-treated sample (NS, not significant) (**D**). Representative electron micrographs are depicted (**E**). Quantification of the number of fibril-containing organelles displaying the collapsed amyloid sheet phenotype. Shown is the average from three independent experiments. An unpaired two-tailed t-test was used to determine whether means are statistically different from the DMSO-treated sample (***p < 0.0001) (E, *lower panel*).

In order to directly assess the role of O-glycosylation in shaping PMEL amyloid morphology, we treated Mel220 cells expressing wildtype human PMEL with the O-glycosylation inhibitor Benzyl-2-acetamido-2-deoxy-α-D-galactopyranoside^31–34^ for four days. This treatment did not affect PMEL expression or ER export (Fig. 9A), but dramatically reduced O-glycosylation measured by the reactivity of PMEL with the O-glycosylation-dependent antibody HMB45 recognizing a sialylated epitope^35^ (Fig. 9B). The inhibition of O-glycosylation did not reduce amyloid formation as judged by CAF accumulation (Fig. 9C) and direct EM analysis (Fig. 9D). Strikingly, however, the inhibition of O-glycosylation dramatically affected amyloid morphology with 82% to 87% of melanosomes displaying the collapsed sheet phenotype (Fig. 9E). Thus, even the wildtype RPT domain largely loses its function if un- or underglycosylated. Taken together, these data strongly argue that O-glycosylation is essential for RPT domain function and that O-glycosylation drives the establishment of the three-dimensional fibrillar sheet structure of melanosomal amyloid.

## Discussion

Electron tomography is a powerful method to unravel the 3D architecture of cellular structures and has been used to visualize amyloid deposits^36–38^ including the structure of melanosomal amyloid^5^. The latter study demonstrated that melanosomal amyloid assembles into a sheet architecture^5^, which has likely evolved to provide a large planar surface area for melanin deposition. Highly reactive melanin precursor molecules adsorb onto these sheets, allowing their accumulation at high local concentrations. This accelerates pigment formation and also sequesters toxic, oxidative compounds in the melanin synthesis pathway^1^. Thus, the overall structure of the PMEL matrix is likely linked to its function in pigmentation biology. It is of considerable interest to understand the mechanisms that shape it.

We identify the highly O-glycosylated RPT domain as the critical factor driving the formation of the PMEL amyloid sheet architecture. Specifically, we demonstrate that without the RPT domain PMEL amyloid does not organize into sheets but instead collapses into a block-like assembly (Fig. 3C,D and Supplementary Fig. S4A–C). Interestingly, previous studies had found no fibrillar material when PMEL-ΔRPT mutants were expressed in HeLa cells, which lack melanosomes. However, wildtype PMEL-transfected HeLa cells can to some extent form fibrils in late endosomal/lysosomal compartments^9,10^. It is possible that these differences are caused by a higher susceptibility of the mutant fibrils to the harsh acidic and proteolytic environment in lysosomes, because they lack potentially protective O-glycosylation. Alternatively, melanocytes may be more permissive for optimal folding of PMEL-ΔRPT than HeLa cells. In any case, ΔRPT block-like assemblies are unlikely to adequately support melanin deposition as less surface area is available for pigment adsorption. However, investigating this in the future will require a pigmented melanoma experimental system actively producing melanin (Mel220 cells are unpigmented). Defective pigment deposition may have significant implications for melanin detoxification. PMEL loss-of-function mutations including the *Smoky* allele in chickens^25,26^ and the *silver* mutation in mice^10^ are associated with reduced pigmentation similar to PMEL knockout mice^39^. This likely reflects reduced melanocyte viability in the absence of functional PMEL^29,40,41^, which is also consistent with the observation that the human *PMEL* locus is under purifying selection^39^. Defective melanin detoxification probably underlies reduced melanocyte viability in *silver* mice and an analogous scenario may apply in cells expressing ΔRPT mutants. If the solid ΔRPT block-like assemblies are less supportive of melanin deposition, toxic pigment precursors^6^ may leak out of melanosomes, causing oxidative damage to cellular proteins and DNA. Thus, the ultimate function of the RPT domain may be to mitigate pigmentation-associated toxicity.

Given this potentially crucial function, it is puzzling that the RPT domain is the only PMEL lumenal domain whose primary amino acid sequence is not conserved (Fig. 4A and Supplementary Fig. S1A). Nevertheless, its deletion from human (Fig. 1B,C), mouse (Fig. 2B,C,E), and frog (compare Fig. 5D and Fig. 7K) PMEL produces identical phenotypes and RPT domains from most species are interchangeable modules (Fig. 7A,B). Thus, despite substantial sequence variation the function of the RPT domain is conserved. Only the fish RPT domains act much less effectively in the context of human and frog PMEL (Fig. 7A–C,J,K). However, even they display measurable functionality in various sequence contexts with 11–28% of melanosomes harboring separated amyloid sheets (Fig. 7A,C,J) compared to 0% of such organelles in the context of ΔRPT mutants (Figs 7J and 8H). Interestingly, melanosomes in zebrafish retinal pigment epithelium (RPE) seem to contain densely packed fibrillar material with relatively small internal cavities^28^, very much unlike the well-separated sheets in mammalian organelles (Fig. 3A)^5^. Thus, fish RPT domains may be less functional even in their native context. This could reflect an intrinsically reduced capability to mediate sheet separation despite proper association with the amyloid. Alternatively, the domains could undergo heavy proteolysis within the amyloid (as the human RPT domain does^9^) resulting in significant shedding of fragments, thereby reducing the effective concentration of RPT domain in the fibrils below the threshold of adequate function. Another possibility is that the fish RPT domains do not efficiently incorporate into the PMEL matrix in the first place.

The sheet architecture of melanosomal amyloid essentially depends on O-glycosylation, as even the wildtype human RPT domain is unable to promote the normal morphology if O-glycosylation is inhibited (Fig. 9). Dense O-glycans can prevent protein-protein interactions. In mucins, for instance, they serve to shield the protein from protease attack^30^. A similar mechanism may underlie the separation of PMEL amyloid sheets. O-glycans pointing away from the fibrillar layers may prevent direct sheet-sheet contact, thereby suppressing their association and collapse into a single block (Supplementary Fig. S4A–C). This would explain why even a randomly selected, O-glycosylated segment completely unrelated in sequence to any known RPT domain can at least partially substitute for the function of the human RPT domain (Fig. 8G,H).

Interestingly, a complete loss of glycosylation does not appear to be necessary for sheet collapse. Both the human (Fig. 1) and the mouse ΔRPT mutant (Fig. 2A–C and Fig. 3) are predicted to retain 11 O-glycosylation sites within MαC but nevertheless display the full phenotype observed in the absence of glycosylation (construct ΔRPTΔGlyc) (Fig. 2D,E)). This raises the question whether RPT domain function depends on the number or on the type of the glycans. In the first case, it would be interesting to know how much glycosylation is needed to establish the characteristic PMEL sheets. In the second case, it would be interesting to assess which particular oligosaccharides are relevant.

Another important question is why the MUC2-derived repeat sequence is only partially but not fully functional as a substitute for an RPT domain (Fig. 8G,H). Given that the MUC2 sequence is only three amino acids shorter than the mouse RPT domain and contains only three predicted O-glycans more than the frog PMEL-A RPT domain, length or number of O-glycans *per se* are probably not the critical factors. However, the predicted O-glycan density on the MUC2 fragment is about twice as high as on PMEL RPT domains. This could interfere with proper binding to the PMEL amyloid and/or cause increased shedding.

Moreover, RPT domains generally have a negative overall charge which is not found on the MUC2 segment. Such a negative charge may aid via electrostatic repulsion in the outward positioning of sialic acid-carrying carbohydrates and thereby support amyloid sheet separation. However, as our inhibitor experiments in Fig. 9E demonstrate, the negative charges carried by aspartate and glutamate residues alone in the un-/underglycosylated human RPT domain are not sufficient to drive proper amyloid sheet separation. Moreover, the RPT domain derived from zebrafish PMEL-A, which carries by far the largest number of negative charges compared to other RPT domains in our set, is by far the least efficient in mediating fibril spacing (Fig. 7A–C). This calls into question whether the three-dimensional organization of PMEL amyloid into sheets is merely a matter of charge. In fact, other aspects of the chemical nature of RPT domain-associated O-glycans may play a more important role. Future experiments employing a PMEL mutant, in which the RPT domain has been replaced by a highly negatively charged, but unglycosylated domain (*i.e*. the counterpart of the PMEL-RPT_MUC2_ chimera with its largely uncharged, but highly glycosylated RPT domain) may more definitively clarify the role of charge in PMEL amyloid structure, but such experiments would have to carefully control for proper incorporation of the domain into the fibrils.

Finally, RPT domain glycosylation may not only be important in indirectly preventing melanin-associated toxicity (see above) but may additionally mitigate amyloid-associated toxicity in a more direct manner. Specifically, glycans could shield potentially harmful interactions between the PMEL amyloid and membranes^42–45^. Alternatively, they could suppress secondary nucleation events, which often drive high amyloid toxicity^46^ and which likely depend on a naked amyloid surface^47^. We have not observed acute toxicity of our ΔRPT mutants in Mel220 cells, but more subtle phenotypes may exist and primary melanocytes may be more sensitive than a tumor cell line. Moreover, oxidative damage can cause membrane disruption and leakage^48^ and might synergize in a melanin-producing organelle with potential direct amyloid toxicity. Isolated cytosolic melanocores not surrounded by a membrane as well as late (but not early) melanosomes with partially disrupted membranes have been observed in melanoma cells^49^. Under such circumstances stable binding of toxic pigment compounds to the amyloid matrix may be particularly critical and mutants lacking the RPT domain may be very poorly protective.

## Material and Methods

### Animals and ethics statement

Maintenance of and experiments on animals were approved by the Geneva Canton ethical regulation authority (authorization GE/82/14 and GE/73/16) and performed according to Swiss law. Corn snakes were bred at the LANE, University of Geneva.

### Cell lines and cell culture

LG2-MEL-220 (Mel220), a human PMEL-deficient melanoma cell line^50^, was grown in IMDM (Sigma)/10% FCS (HyClone) containing non-essential amino acids (Gibco), GlutaMax (Gibco) and penicillin/streptomycin (Gibco). PMEL transfectants were grown in medium additionally containing 2 mg/ml G418 (Gibco).

### Vector constructs and PMEL expression

Mouse PMEL was cloned by a HiFi Platinum Taq-driven PCR (Invitrogen) using primer pair 5′-TTTGGCTGCTGGCAAGAGGACC-3′/5′-CCCAGGAAATCCACGGTGCC-3′ and YUMM1.1 cDNA (murine melanoma cDNA kindly provided by Dr. J. Jacox) as a template. To clone *Pantherophis guttatus* (corn snake) PMEL, total RNA was obtained from the skin of one individual corn snake as described^51^ and cDNA was synthesized (AffinityScript Multi Temperature cDNA Synthesis Kit (Agilent)). Snake PMEL was then amplified by a HiFi Platinum Taq-driven PCR (Invitrogen) using primer pair 5′-ATGTCACGGATCTGGTTCCTATGGG-3′/5′-GTATCTCCCCACCCAGGCAGGC-3′. This lead to the cloning of two cDNAs corresponding to two different alleles of the single corn snake PMEL gene (see Supplementary Fig. S2 for details). Both alleles aligned well with two overlapping Pantherophis guttatus-derived contigs obtained from the Reptilian Transcriptomes Database 2.0^52^ (*contig_IlluminaI_7640* and *contig_454_10910*). The two contigs are spanning the entire open reading frame. Both mouse and snake PMEL were captured into pCR 2.1-TOPO using the TOPO TA Cloning Kit (Invitrogen). The cloned mouse PMEL (MH882516.1), snake PMEL allele A (MH882514.1), and snake PMEL allele B (MH882515.1) sequences were deposited in GenBank.

The cDNA encoding the chicken *Dominant White* PMEL allele in pcDNA3.1 + /C-(K)DYK (Clone ID: OGa27055C) was purchased from Genscript. The three amino acid transmembrane insertion, which renders this allele pathogenic^25,26^, was removed using a standard QuikChange mutagenesis with primer pair 5′-CCGTGGGGCTGCTCCTCATGGCCGCTGC-3′/5′-GCAGCGGCCATGAGGAGCAGCCCCACGG-3′. Xenopus laevis PMEL-A in pCMV-SPORT6.ccdb (Clone ID: 6952781) and PMEL-B in pCMV-SPORT6 (Clone ID: 4959771) were purchased from Dharmacon. Zebrafish PMEL-A in pExpress-1 (Clone ID: BC117628) and PMEL-B (Clone ID: BC116462) were purchased from Transomic. The single EcoRI cleavage site in zebrafish PMEL-A was silently modified using an overlap extension PCR with inner primers 5′-TTTGCACGTTATCGTTCATGGAACTCACAGATGTATCCGGTCTGGAGG-3′/5′-CCTCCAGACCGGATACATCTGTGAGTTCCATGAACGATAACGTGCAAA-3′ and appropriate outer primers (Supplementary Table S1). Mouse PMEL and all non-mammalian PMEL genes were amplified in a HiFi Platinum Taq-driven PCR (Invitrogen) using primer pairs listed in Supplementary Table S1 and cloned into pBMN-IRES-neo^19^ as EcoRI-EcoRI fragments.

Chimeric RPT domain-swapping PMEL mutants were cloned by overlap extension PCR combining three PCR fragments (N-terminal, RPT domain-containing middle, and C-terminal segment) in a three-step HiFi Platinum Taq-driven reaction (see Supplementary Table S2 for sequence details). Fragments were synthesized using the primer pairs given in Supplementary Table S2. All chimeric mutants that are based on human PMEL contain the N-terminus of human PMEL until and including residue Cys-301, followed by a non-human RPT domain, which starts with the first amino acid following this highly conserved cysteine residue and spanning up until the P5 residue of the proprotein convertase cleavage motif (nomenclature as published^53^), which is generally present in PMEL. This is followed by the entire C-terminal sequence of human PMEL, starting with the P4 residue of the cleavage motif (Leu-466). Thus, all chimeric constructs share the same core of the human proprotein convertase cleavage motif (residues P4 through P1)^53^. An analogous strategy was employed to clone construct xenopus laevis PMEL-A-RPT_DR-A_ (see Supplementary Table S2 for sequence details and primers).

A human MUC2-derived fragment was cloned by RT-PCR using primer pair 5′-GCAGTGTGATGTCTCTGTTGGGTTC-3′/5′-GGGAACATCAGGATACATGGTGGC-3′ and captured by TA cloning. The resulting vector was used as a template to PCR-amplify a smaller MUC2 fragment using primer pair 5′-GTTGCTGGCCCATGGATAAGTGTATC-3′/5′-GTGGAAGGGTGGTAGTGCTGGC-3′, and this smaller fragment was again captured by TA cloning. This vector was used as a template to synthesize the middle segment of construct PMEL-RPT_MUC2_. This construct was synthesized analogously to the RPT-chimeric PMEL constructs using the primer pairs listed in Supplementary Table S2.

Xenopus laevis PMEL-A mutant ΔRPT (Δ308–592) was cloned by a standard QuikChange mutagenesis using Xenopus PMEL-A in pBMN-IRES-neo as a template together with primer pair 5′-CTACACCATGTGGCGTGGTTATTGCAAAGC-3′/5′-GCTTTGCAATAACCACGCCACATGGTGTAG-3′. Mouse PMEL mutants ΔRPT (Δ315–402) and ΔRPTΔGlyc (Δ304–429/S299G/S302P/S303G) were cloned by a standard QuikChange mutagenesis using mouse PMEL in pBMN-IRES-neo as a template together with primer pairs 5′-GATGGCTACATGCCAGAGGGTCCAGATGC-3′/5′-GCATCTGGACCCTCTGGCATGTAGCCATC-3′ and 5′-GCCATTCCTCTTGTTGGCTGTGGTCCCGGGATAATGCTTGTGAAG-3′/5′-CTTCACAAGCATTATCCCGGGACCACAGCCAACAAGAGGAATGGC-3′, respectively.

All vectors were sequenced before retroviral transduction^13^ into Mel220 cells and selection with 2 mg/ml G418 (Gibco). The human PMEL constructs wt-PMEL and ΔRPT in pBMN-IRES-neo have been described^13,19^.

### Antibodies and Chemicals

I51 is a rabbit polyclonal antiserum recognizing the human and mouse PMEL CAF^7^. The rabbit polyclonal antisera 6777 and 6778 also recognize the human and mouse CAF and were raised by Genscript to a peptide with sequence SSAFTITDQVPFSVSVSQLRC (human PMEL 204–223 appended with a cysteine) conjugated to KLH. NKI-beteb (Abcam) is a mouse monoclonal antibody recognizing a conformation-sensitive epitope within PMEL^19,54^. HMB45 (NeoMarkers) is a mouse monoclonal antibody recognizing the sialylated RPT domain^35^. E-7 (Santa Cruz) (sc-377325) is a mouse monoclonal antibody recognizing the PMEL N-terminus^8^. EP4863(2) (Abcam) and EPR4864 (Abcam) are rabbit monoclonal antibodies recognizing the PMEL N-terminus (NTF)^13^ and the PMEL C-terminus, respectively. HRP- and fluorophore-labeled secondary antibodies were purchased from Jackson ImmunoResearch and Molecular Probes.

The O-glycosylation inhibitor Benzyl-2-acetamido-2-deoxy-α-D-galactopyranoside^31^ was purchased from Sigma Aldrich. For each experiment, a 100 mg/ml stock solution was freshly prepared in DMSO and Mel220 cells expressing human PMEL were treated for four days at a final concentration of 0.4 mg/ml. Benzyl-2-acetamido-2-deoxy-α-D-galactopyranoside had originally been described as a T synthase inhibitor^34^, but later work demonstrated that the compound blocked carbohydrate maturation mainly by inhibiting sialylation^32,33^.

### Western blotting, flow cytometry, and RT-PCR

Total membranes were prepared as described^55^. Briefly, 5 × 10^6^ cells were resuspended in 1 ml 10 mM Tris-HCl pH 7.4 containing protease inhibitor (Complete, Roche) and incubated for 10 min on ice. Lysed cells were Dounce homogenized with 30 strokes (“tight” pestle) and centrifuged at 800 g/4 °C/10 min. The resulting supernatant was spun at 45,000 rpm/4 °C using the TLA-55 rotor in a Beckman Optima TL ultracentrifuge. The pellet was lysed in PBS/1% SDS/1% β-mercaptoethanol for 10 min at RT followed by 10 min at 95 °C and subjected to SDS-PAGE. Western blotting was carried out as described^56^.

Flow cytometry was performed as described^57^ using the antibodies NKI-beteb or EP4863(2) at a concentration of 1: 10 and 1: 100, respectively, followed by Alexa647- or Alexa488-conjugated secondary antibodies on an Accuri C6 flow cytometer.

Total RNA was extracted from Mel220 transfectants using the RNeasy Mini Kit (Qiagen) and cDNA was prepared using the AffinityScript Multi Temperature cDNA Synthesis Kit (Agilent). Actin-specific primers have been described^58^. To amplify a fragment containing the full open reading frame of human and non-human PMEL, pBMN-IRES-neo-specific vector primers were used (5′-GACCACCCCCACCGCCCTC-3′/5′-GAATGCTCGTCAAGAAGACAGGGC-3′) in a standard Taq-driven PCR (annealing temperature 64.8 °C/extension time 2 min 51 sec/28 cycles).

### Electron microscopy and tomography

Cells in Petri dishes were fixed for one hour using 2.5% glutaraldehyde in 0.1 M sodium cacodylate buffer pH 7.4 containing 2% sucrose. Buffer-rinsed cells were scraped in 1% gelatin and spun down in 2% agar. Chilled blocks were trimmed and post-fixed in 1% osmium tetroxide for one hour. Next, the samples were rinsed three times in sodium cacodylate rinse buffer and post-fixed in 1% osmium tetroxide for one hour. Then they were rinsed and en-bloc stained in aqueous 2% uranyl acetate for one hour, rinsed again, dehydrated in an ethanol series, and infiltrated with Embed 812 (Electron Microscopy Sciences) resin. The samples were placed in silicone molds and baked at 60 °C for 24 hours. Hardened blocks were sectioned using a Leica UltraCut UC7. 60 nm sections were collected on Formvar-coated nickel grids and 250 nm sections were collected on copper slot grids and stained using 2% uranyl acetate and lead citrate. 60 nm sections were viewed using a FEI Tecnai Biotwin TEM at 80 kV. Images were collected using a Morada CCD camera and iTEM (Olympus) software. Fibril-containing organelles in 15 arbitrarily chosen cells in one view field per sample were counted as described previously^13^. For electron tomography, 15 nm fiducial gold particles were added on sections before imaging. The dual-axis tilt series was collected using a FEI Tecnai TF20 at 200 kV TEM equipped with a field emission gun. Images were recorded using SerialEM software (UC, Boulder) and a FEI Eagle 4Kx4K CCD camera. Tilting range was from −60° to 60° at 1° increments. Tomogram reconstruction, segmentation, and modeling were performed using IMOD (UC, Boulder).

## Supplementary information

Movie S1

Movie S2

Supplementary Material

## Data Availability

The datasets generated during and/or analyzed during the current study are available from the corresponding author on reasonable request.
