# Supplementary material for: Repeat domain-associated O-glycans govern PMEL fibrillar sheet architecture
Source: Sci Rep. 2019 Apr 15;9:6101. doi: 10.1038/s41598-019-42571-6 (PMC6465243; doi:10.1038/s41598-019-42571-6)
Supplement: Supplementary file 3 — Supplementary Material [file 41598_2019_42571_MOESM3_ESM.pdf]

Supplementary Material

Repeat domain-associated O-glycans govern PMEL fibrillar sheet architecture

Morven Graham[a,1], Athanasia C. Tzika[c,1], Susan M. Mitchell[b,1], Xinran Liu[a], and Ralf M. Leonhardt[b,*]

From the [a]Department of Cell Biology and the [b]Department of Immunobiology, Yale University School of Medicine, 300 Cedar Street, New Haven CT 06519, USA and the [c]Department of Genetics & Evolution, Laboratory of Artificial & Natural Evolution (LANE), Sciences III Building, 1211 Geneva 4, Switzerland

[1] equal contributions

[*] Address correspondence to: Ralf M. Leonhardt, Ph.D., Boehringer Ingelheim RCV GmbH & Co KG, Cancer Immunology & Immune Modulation, Dr. Boehringer Gasse 5-11, A-1121 Vienna, Austria,

Tel. +43 1 80105-9310;

E-mail: Ralf.Leonhardt@boehringer-ingelheim.com

SUPPLEMENTARY TABLE LEGENDS

Suppl. Table S1. Primers used for transfer of PMEL genes into pBMN-IRES-neo. The indicated primers were used to PCR-amplify non-human PMEL genes before cloning as EcoRI-EcoRI fragments into vector pBMN-IRES-neo. EcoRI sites are labeled in red. Sequences that are part of the respective non-human PMEL genes are labeled in green. Sequences between the EcoRI sites and the non-human PMEL genes (labeled in black) were chosen to exactly match the respective sequences in the human PMEL expression construct.

Suppl. Table S2. Primers used to clone RPT domain swapping mutants. The indicated primers were used to clone chimeric PMEL constructs by overlap extension PCR (HiFi Platinum Taq (Invitrogen)). For each construct, three PCR fragments were synthesized, corresponding to the N-terminus (primer pair *N-term. forward/N-term. reverse*), the middle segment (primer pair *Middle forward/Middle reverse*), and the C-terminus (primer pair *C-term. forward/C-term. reverse*) of the chimeric mutant. Human PMEL in pBMN-IRES-neo was used as a template to synthesize all N-terminal and C-terminal fragments except for construct Xenopus PMEL-A-RPT_{DR-A}, for which the template was Xenopus PMEL-A in pBMN-IRES-neo. The template DNA for the middle segment was the respective non-human PMEL gene in pBMN-IRES-neo or a vector containing a fragment derived from the MUC2 gene. After the synthesis of the three PCR fragments, the N-terminal fragment and the middle fragment were combined by overlap extension PCR (outer primers were added to the reaction only after six PCR cycles). Subsequently, this combined fragment was joined to the C-terminal fragment by overlap extension PCR (again, outer primers were added to the reaction only after six PCR cycles) to synthesize the entire open reading frame of the mutant. This combined fragment was cleaved with EcoRI and cloned as an EcoRI-EcoRI fragment into pBMN-IRES-neo. Vector-specific primers in Supplementary Table S2 are shown in gray. Human PMEL-specific sequences are shown in black. Non-human PMEL- or MUC2-specific sequences are shown in color. The nucleotide causing the C1395S mutation in the MUC2 segment of construct PMEL-RPT_{MUC2} (designed to

suppress a potential unwanted disulfide bridge between Cys-301 (in human PMEL) and Cys-1395 (in human MUC2), which would be only five amino acids apart in this construct (Supplementary Fig. S4B)) is shown in bold on green background.

SUPPLEMENTARY FIGURE LEGENDS

Suppl. Figure S1. Multiple sequence alignment of PMEL genes derived from different species. (A) PMEL genes derived from mammal (Homo sapiens, Mus musculus), bird (Gallus gallus), reptile (Pantherophis guttatus), amphibian (Xenopus laevis), and fish (Danio rerio) origin were aligned using the *Clustal Omega Multiple Sequence Alignment* program (1.2.4). Individual PMEL domains are highlighted with unique colors (NTF, N-terminal fragment; CAF, core amyloid fragment; PKD, polycystic kidney disease-like domain; RPT, repeat domain; KLD, Kringle-like domain; TMD, transmembrane domain; C-term., cytosolic C-terminus). Identical residues, defined as positions that are occupied by the same amino acid in all nine PMEL genes, are marked with an asterisk. Positions with mere similarity are indicated with a colon or a period. Percent identity within individual PMEL domains was calculated as the number of identical residues in the respective domain divided by the length of the domain in human PMEL and is shown as a bar diagram in Fig. 4A.

Suppl. Figure S2. Identification and cloning of two alleles of the single corn snake PMEL gene. (A) Full-length corn snake PMEL cDNA derived from the skin of one individual snake was amplified with appropriate primers and the entire open reading frame was sequenced. In four positions two overlapping peaks (SNP1, SNP2, SNP3, and SNP5), and in a fifth position a series of overlapping peaks corresponding to an in-frame three-nucleotide deletion in a part of the sequenced material (SNP4) was observed, suggesting the mix of two different alleles. All five SNPs were confirmed in a second independent RT-PCR, thus, excluding that they merely reflected PCR errors. Sequence analysis revealed that SNP5 would be silent, encoding Gly-693 in both alleles. SNP4 reflected the deletion of Val-477 in one but not the other allele. SNP1 (Ser-358 / Phe-358), SNP2 (Ala-374 / Thr-374), and SNP3 (Asp-408 / Asn-408) reflected missense point mutations. **(B)** In order to determine the exact sequence of the two corn snake PMEL alleles, we designed forward primers specifically amplifying only the allele encoding Ser-358_{TCT} or only the allele encoding Phe-358_{TTT}. Likewise, reverse primers were designed specifically

amplifying only the allele containing the Gly-693$_{GGA}$ codon or only the allele containing the Gly-693$_{GGC}$ codon. An RT-PCR was performed using all four primer combinations. Only the two primer combinations Ser-358$_{TCT}$ / Gly-693$_{GGA}$ and Phe-358$_{TTT}$ / Gly-693$_{GGC}$ produced significant product, indicating that allele A contained the Ser-358$_{TCT}$ and Gly-693$_{GGA}$ SNPs while allele B contained the Phe-358$_{TTT}$ and Gly-693$_{GGC}$ SNPs. **(C)** The PCR products from Supplementary Fig. S2B were sequenced in order to determine the nucleotide sequence at the SNP2, SNP3, and SNP4 positions in corn snake PMEL alleles A and B. This resulted in the full characterization of both alleles (allele A = Ser-358$_{TCT}$ / Thr-374$_{ACA}$ / Asp-408$_{GAT}$ / Val-477$_{GTA}$ / Gly-693$_{GGA}$ and allele B = Phe-358$_{TTT}$ / Ala-374$_{GCA}$ / Asn-408$_{AAT}$ / ΔVal-477 / Gly-693$_{GGC}$). **(D)** All differences in amino acid sequence between the two alleles (non-silent SNPs) fall inside the RPT domain. Asparagine in position 408 is a predicted site for N-glycosylation. Both corn snake PMEL alleles were cloned.

Suppl. Figure S3. In contrast to PMEL-RPT$_{XL-A}$, most melanosomes in PMEL-RPT$_{DR-A}$-expressing cells display the collapsed sheet phenotype. (A-C) Low magnification images of the EM analysis in Fig. 7B showing many individual melanosomes. Note that the majority of melanosomes in wildtype human PMEL (A) and PMEL-RPT$_{XL-A}$ (B)-expressing Mel220 cells displays the normal amyloid sheet morphology, while Mel220 cells expressing construct PMEL-RPT$_{DR-A}$ (C) contain mostly organelles harboring collapsed sheets. This result is quantified in Fig. 7C. **(D)** Example EM images of organelles with well-separated amyloid sheets (*left panels*) or collapsed sheets (*right panels*) as observed in cells expressing constructs PMEL-RPT$_{XL-A}$, PMEL-RPT$_{XL-B}$, PMEL-RPT$_{DR-A}$, and PMEL-RPT$_{DR-B}$. That both types of morphologies are found in all four mutants shows that the differences in amyloid morphology between the mutants are primarily quantitative not qualitative in nature.

Suppl. Figure S4. RPT domain-associated glycans mediate proper PMEL amyloid sheet spacing. (A) Domain organization of the PMEL Mα fragment. Three N-linked glycans in the N-terminal NTF

region and dense O-glycosylation in the RPT domain are indicated (NTF, N-terminal fragment; CAF, core amyloid fragment; PKD, polycystic kidney disease-like domain; RPT, repeat domain). **(B)** Spacing of PMEL amyloid sheets is mediated by RPT domain-associated glycans. **(C)** In the absence of the RPT domain normal sheet-sheet separation breaks down and amyloid sheets collapse into a block-like assembly.

MOVIES

Movie S1. Electron tomography of wildtype mouse PMEL-containing melanosomes.

Movie S2. Electron tomography of mouse ΔRPT-containing melanosomes.

Supplementary Table S1, ***Primers used for transfer of PMEL genes into pBMN-IRES-neo.***

mouse PMEL<br>(mus musculus)	5′-ttgaattccaccATGGGTGTCCAGAGAAGGAGC-3′ *(forward)*<br>5′-aagaattcgccgcTCAGACCTGCTGTCCACTGAGG-3′ *(reverse)*
chicken PMEL<br>(gallus gallus)	5′-ttgaattccaccATGCGGTTGCACGGGGCC-3′ *(forward)*<br>5′-aagaattcgccgcTTAGACAGCGTTGGCACGCAGCA-3′ *(reverse)*
Corn snake PMEL<br>(Pantherophis guttatus)	5′-ttgaattccaccATGTCACGGATCTGGTTCCTATGG-3′ *(forward)*<br>5′-aagaattcgccgcTTAGATGACGTTGCCACTGAGCA-3′ *(reverse)*
frog PMEL-A<br>(xenopus laevis)	5′-ttgaattccaccATGAAGGGTTTATGCTGGCTGG-3′ *(forward)*<br>5′-aagaattcgccgcTCACACTACCCGGCCATTCAAAAG-3′ *(reverse)*
frog PMEL-B<br>(xenopus laevis)	5′-ttgaattccaccATGAAGGGGGTATACGGTCTGGTG-3′ *(forward)*<br>5′-aagaattcgccgcTCACACAACCCGGCCATTCAA-3′ *(reverse)*
zebrafish PMEL-A<br>(danio rerio)	5′-ttgaattccaccATGTGGACATCTCTCATCTTCCTC-3′ *(forward)*<br>5′-aagaattcgccgcTCACACCACGCGTCCCAGCA-3′ *(reverse)*
zebrafish PMEL-B<br>(danio rerio)	5′-ttgaattccaccATGAAGCTATTCTCTACAATATTCATATTAT-3′ *(forward)*<br>5′-aagaattcgccgcTCACACTACTCCTTTTTTCTGATCC-3′ *(reverse)*

Supplementary Table S2, ***Primers used to clone RPT domain swapping mutants.***

All chimeras (vector primer)	5'-GACCACCCCCACCGCCCTC-3' *(N-term. forward)*
All chimeras (vector primer)	5′-GAATGCTCGTCAAGAAGACAGGGC-3′ *(C-term. reverse)*
**PMEL-RPT$_{MM}$**<br>(human PMEL$_{1-301}$<br>mouse PMEL$_{301-431}$<br>human PMEL$_{466-661}$)	5′-CCGGGACTGGGGAGGAACCACAGGAGGTGAGAGGAATGGCAGC-3′ *(N-term. reverse)*<br>5′-GCTGCCATTCCTCTCACCTCCTGTGGTTCCTCCCCAGTCCCGG-3′ *(Middle forward)*<br>5′-CAGGGGGACTTGTCTCTTCACCAGCATTATGGTGTCGGTGTCATCCAGTAG-3′ *(Middle reverse)*<br>5′-CTACTGGATGACACCGACACCATAATGCTGGTGAAGAGACAAGTCCCCCTG-3′ *(C-term. forward)*
**PMEL-RPT$_{GG}$**<br>(human PMEL$_{1-301}$<br>chicken PMEL$_{308-550}$<br>human PMEL$_{466-661}$)	5′-AGGGGGTGCGGAGGTGCCACAGGAGGTGAGAGGAATGGCAGC-3′ *(N-term. reverse)*<br>5′-GCTGCCATTCCTCTCACCTCCTGTGGCACCTCCGCACCCCCT-3′ *(Middle forward)*<br>5′-CAGGGGGACTTGTCTCTTCACCAGCAGCAGCGGCTCCGCTGT-3′ *(Middle reverse)*<br>5′-ACAGCGGAGCCGCTGCTGCTGGTGAAGAGACAAGTCCCCCTG-3′ *(C-term. forward)*
**PMEL-RPT$_{PG-A}$**<br>(human PMEL$_{1-301}$<br>snake PMEL allele A$_{306-506}$<br>human PMEL$_{466-661}$)	5′-AATGGGTCTTCTGAAGTACTGCCTCCACAGGAGGTGAGAGGAATGGCAGC-3′ *(N-term. reverse)*<br>5′-GCTGCCATTCCTCTCACCTCCTGTGGAGGCAGTACTTCAGAAGACCCATT-3′ *(Middle forward)*<br>5′-CAGGGGGACTTGTCTCTTCACCAGGAGCTGCACCACATCTCCTGCG-3′ *(Middle reverse)*<br>5′-CGCAGGAGATGTGGTGCAGCTCCTGGTGAAGAGACAAGTCCCCCTG-3′ *(C-term. forward)*
**PMEL-RPT$_{PG-B}$**<br>(human PMEL$_{1-301}$<br>snake PMEL allele B$_{306-505}$<br>human PMEL$_{466-661}$)	5′-AATGGGTCTTCTGAAGTACTGCCTCCACAGGAGGTGAGAGGAATGGCAGC-3′ *(N-term. reverse)*<br>5′-GCTGCCATTCCTCTCACCTCCTGTGGAGGCAGTACTTCAGAAGACCCATT-3′ *(Middle forward)*<br>5′-CAGGGGGACTTGTCTCTTCACCAGGAGCTGCACCACATCTCCTGCG-3′ *(Middle reverse)*<br>5′-CGCAGGAGATGTGGTGCAGCTCCTGGTGAAGAGACAAGTCCCCCTG-3′ *(C-term. forward)*
**PMEL-RPT$_{XL-A}$**<br>(human PMEL$_{1-301}$<br>xenopus PMEL-A$_{307-594}$<br>human PMEL$_{466-661}$)	5′-GTCTGCTGTTGGAACGGTACTGCCACAGGAGGTGAGAGGAATGGCAGC-3′ *(N-term. reverse)*<br>5′-GCTGCCATTCCTCTCACCTCCTGTGGCAGTACCGTTCCAACAGCAGAC-3′ *(Middle forward)*<br>5′-CAGGGGGACTTGTCTCTTCACCAGAACCACAACCTCCTCTGTGGCAG-3′ *(Middle reverse)*<br>5′-CTGCCACAGAGGAGGTTGTGGTTCTGGTGAAGAGACAAGTCCCCCTG-3′ *(C-term. forward)*
**PMEL-RPT$_{XL-B}$**<br>(human PMEL$_{1-301}$<br>xenopus PMEL-B$_{307-507}$<br>human PMEL$_{466-661}$)	5′-TGTGGCTACTGGAGCAGTACTGCCACAGGAGGTGAGAGGAATGGCAGC-3′ *(N-term. reverse)*<br>5′-GCTGCCATTCCTCTCACCTCCTGTGGCAGTACTGCTCCAGTAGCCACA-3′ *(Middle forward)*<br>5′-CAGGGGGACTTGTCTCTTCACCAGAACCACAACCTCCTCGGCAGC-3′ *(Middle reverse)*<br>5′-GCTGCCGAGGAGGTTGTGGTTCTGGTGAAGAGACAAGTCCCCCTG-3′ *(C-term. forward)*
**PMEL-RPT$_{DR-A}$**<br>(human PMEL$_{1-301}$<br>zebrafish PMEL-A$_{291-613}$<br>human PMEL$_{466-661}$)	5′-TGACTGCTGGAGTTGGATTGACTGGACAGGAGGTGAGAGGAATGGCAGC-3′ *(N-term. reverse)*<br>5′-GCTGCCATTCCTCTCACCTCCTGTCCAGTCAATCCAACTCCAGCAGTCA-3′ *(Middle forward)*<br>5′-CAGGGGGACTTGTCTCTTCACCAGGATTAGTGCGGCCTGAGTTGCTTC-3′ *(Middle reverse)*<br>5′-GAAGCAACTCAGGCCGCACTAATCCTGGTGAAGAGACAAGTCCCCCTG-3′ *(C-term. forward)*
**PMEL-RPT$_{DR-B}$**<br>(human PMEL$_{1-301}$<br>zebrafish PMEL-B$_{295-408}$<br>human PMEL$_{466-661}$)	5′-ACTGGAGGATTCGGTGGACCTGAACAGGAGGTGAGAGGAATGGCAGC-3′ *(N-term. reverse)*<br>5′-GCTGCCATTCCTCTCACCTCCTGTTCAGGTCCACCGAATCCTCCAGT-3′ *(Middle forward)*<br>5′-CAGGGGGACTTGTCTCTTCACCAGCGACACATGGGCTTGGTTTTCAA-3′ *(Middle reverse)*<br>5′-TTGAAAACCAAGCCCATGTGTCGCTGGTGAAGAGACAAGTCCCCCTG-3′ *(C-term. forward)*
**PMEL-RPT$_{MUC2}$**<br>(human PMEL$_{1-301}$<br>human MUC2$_{1390-1517/C1395S}$<br>human PMEL$_{466-661}$)	5′-TGATAC**T**CTTATCCATGGGCCAGCAGGAGGTGAGAGGAATGGCAGC-3′ *(N-term. reverse)*<br>5′-GCTGCCATTCCTCTCACCTCCTGCTGGCCCATGGATAAG**A**GTATCA-3′ *(Middle forward)*<br>5′-CAGGGGGACTTGTCTCTTCACCAGAAGGGTGGTAGTGCTGGC-3′ *(Middle reverse)*<br>5′-GCCAGCACTACCACCCTTCTGGTGAAGAGACAAGTCCCCCTG-3′ *(C-term. forward)*
**PMEL-MαC$_{DR-A}$**<br>(human PMEL$_{1-234}$<br>zebrafish PMEL-A$_{225-613}$<br>human PMEL$_{466-661}$)	5′-CTGAAGGCCACGGCTCGGTTTCTCAGGAAGTGCTTGTTCCCTCC-3′ *(N-term. reverse)*<br>5′-GGAGGGAACAAGCACTTCCTGAGAAACCGAGCCGTGGCCTTCAG-3′ *(Middle forward)*<br>5′-CAGGGGGACTTGTCTCTTCACCAGGATTAGTGCGGCCTGAGTTGCTTC-3′ *(Middle reverse)*<br>5′-GAAGCAACTCAGGCCGCACTAATCCTGGTGAAGAGACAAGTCCCCCTG-3′ *(C-term. forward)*
**Xenopus PMEL-A-RPT$_{DR-A}$**<br>(xenopus PMEL-A$_{1-306}$<br>zebrafish PMEL-A$_{291-613}$<br>xenopus PMEL-A $_{595-803}$)	5′-TGACTGCTGGAGTTGGATTGACTGGACATGGTGTAGTGGGAATGGCAGC-3′ *(N-term. reverse)*<br>5′-GCTGCCATTCCCACTACACCATGTCCAGTCAATCCAACTCCAGCAGTCA-3′ *(Middle forward)*<br>5′-CTCAGGAGCTTGACGCTTTGCAATGATTAGTGCGGCCTGAGTTGCTTC-3′ *(Middle reverse)*<br>5′-GAAGCAACTCAGGCCGCACTAATCATTGCAAAGCGTCAAGCTCCTGAG-3′ *(C-term. forward)*

A

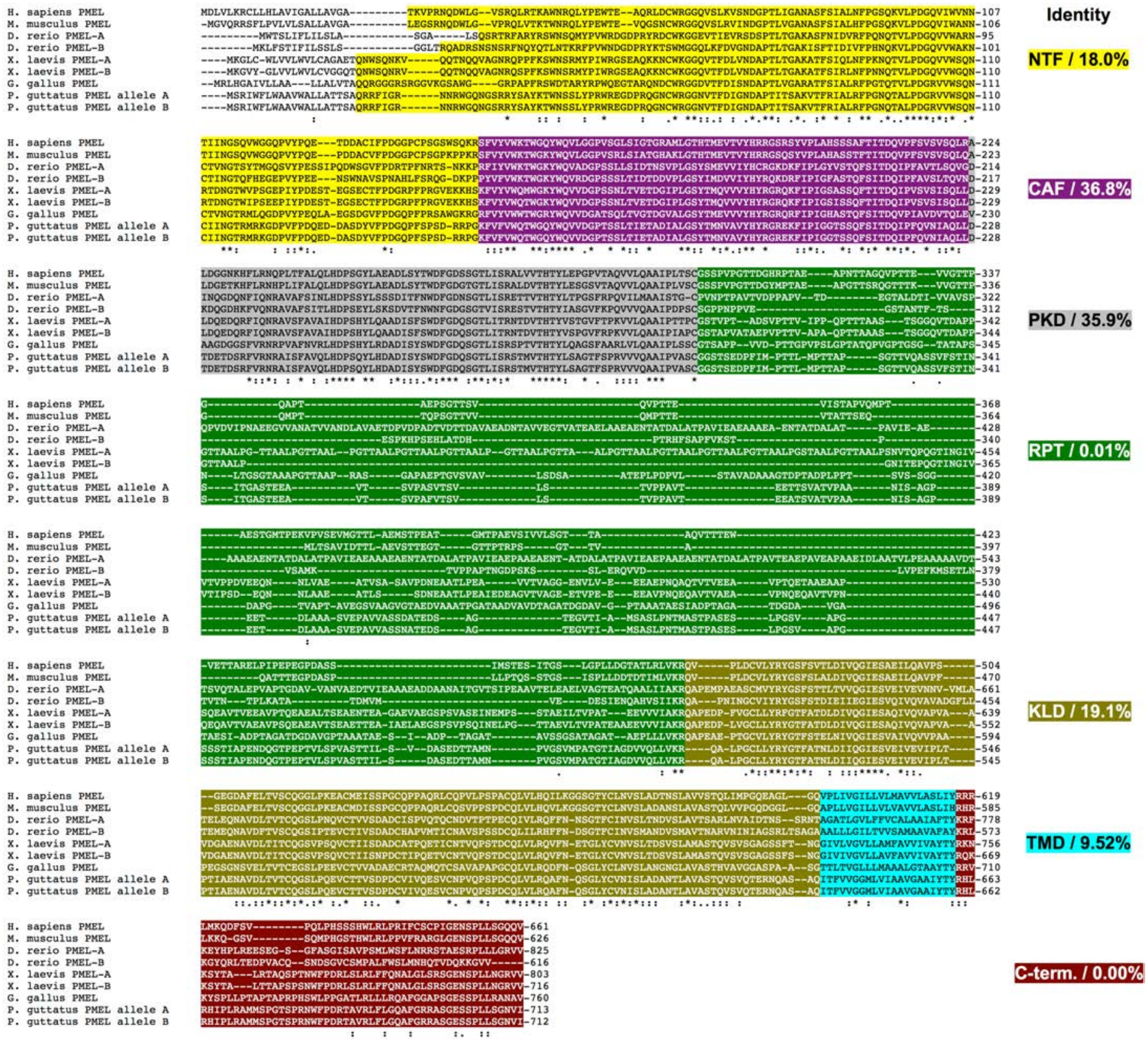

Supplemental Figure S1

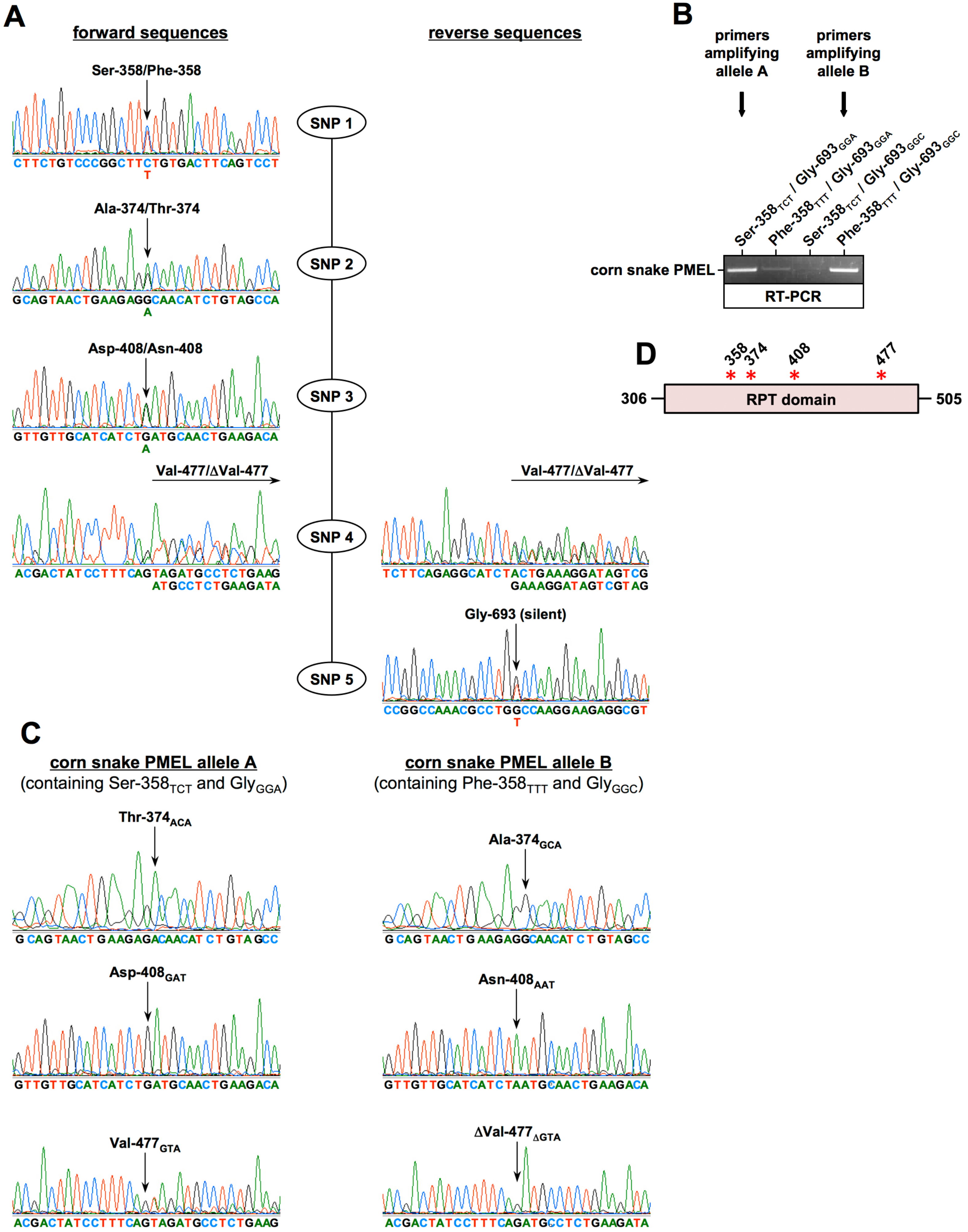

Supplemental Figure S2

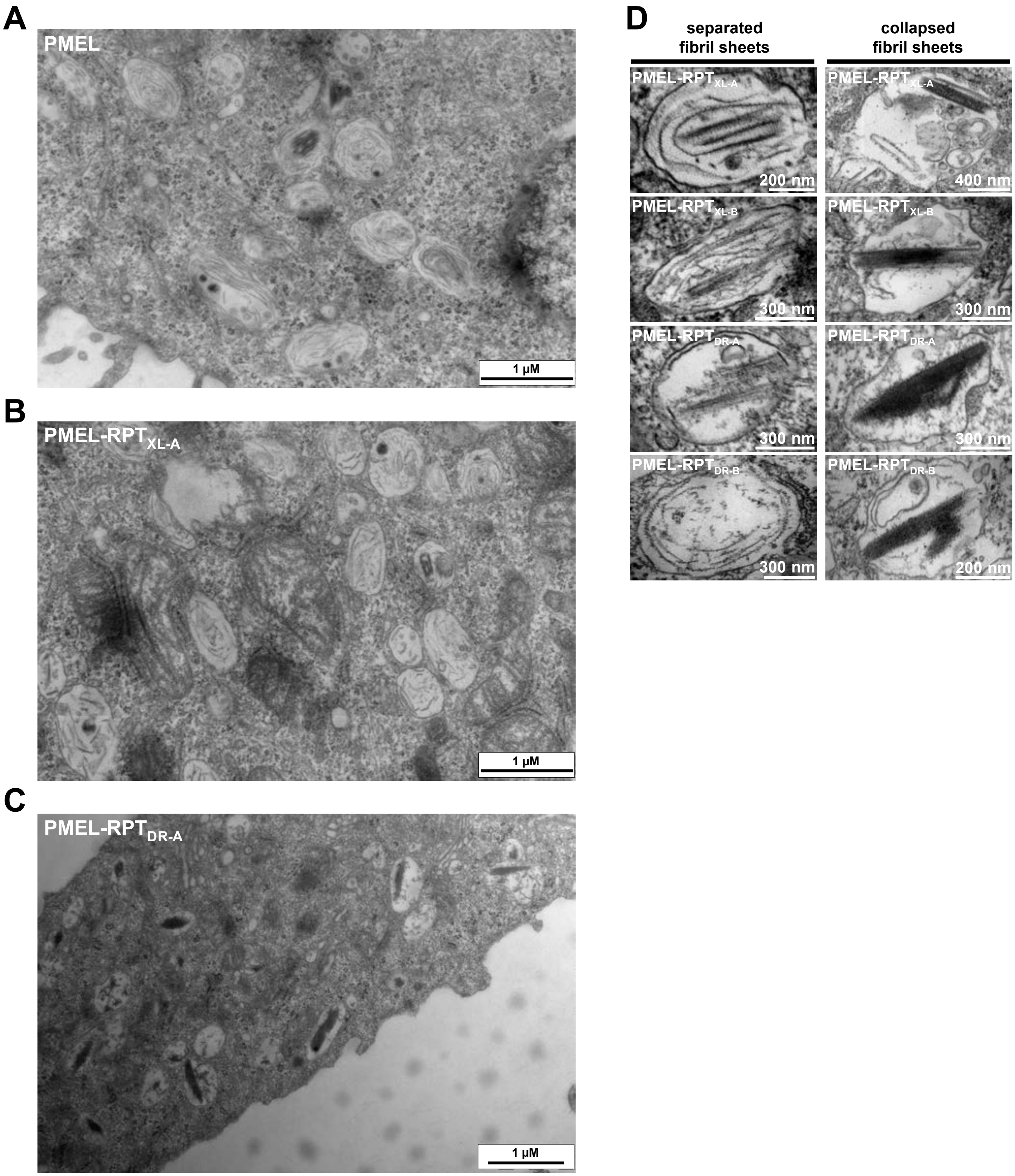

Supplemental Figure S3

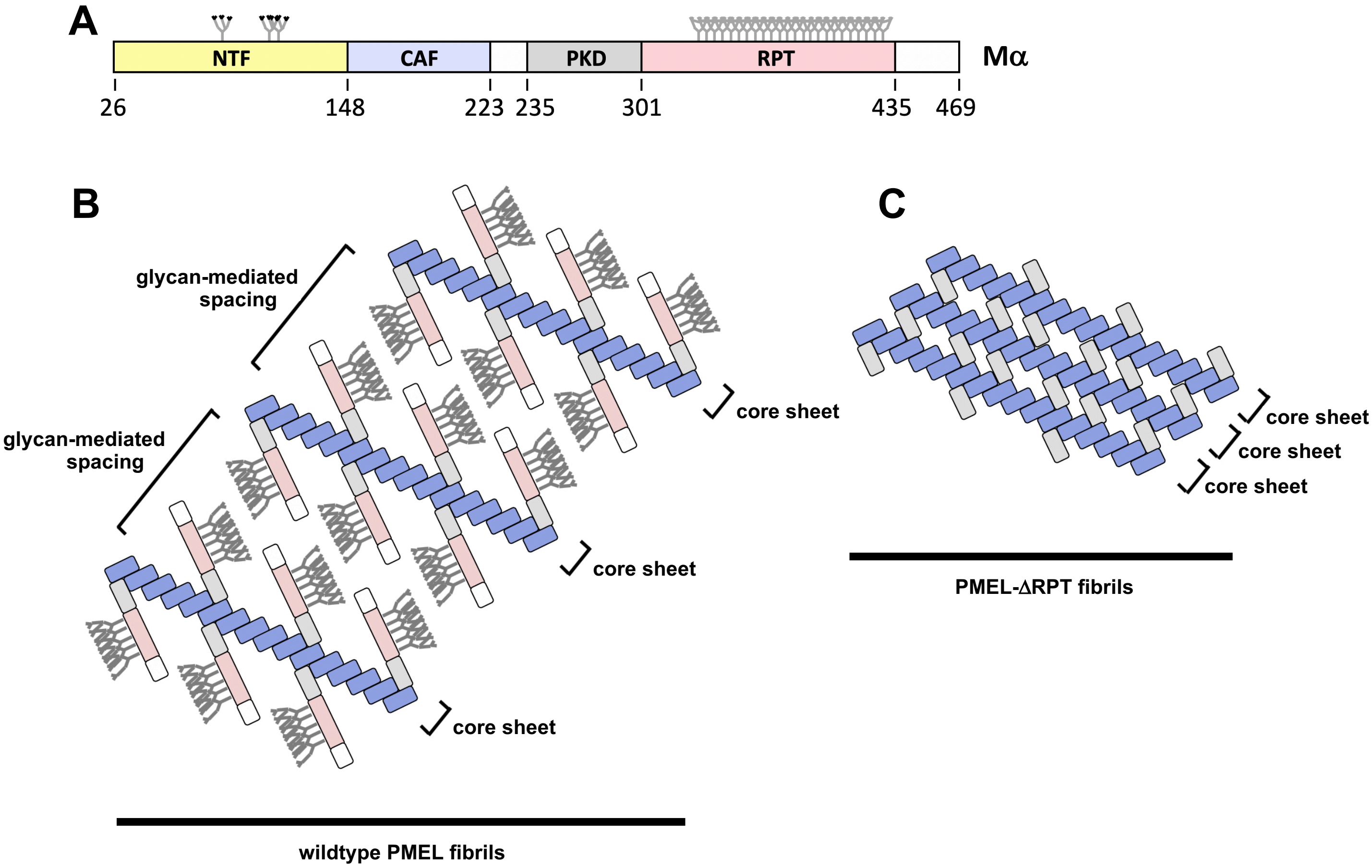

Supplemental Figure S4
